# Social protection and the level and inequality of child mortality in 101 low- and middle-income countries: A statistical modelling analysis

**DOI:** 10.7189/jogh.11.04067

**Published:** 2021-10-23

**Authors:** Zhihui Li, Xinyan Zhou, Shuyao Ran, Fernando C Wehrmeister

**Affiliations:** 1 Vanke School of Public Health, Tsinghua University, Beijing, China; 2 Department of Epidemiology, Mailman School of Public Health, Columbia University, New York, NY, USA; 3 Department of Preventive Medicine, Keck School of Medicine, University of Southern California, Los Angeles, CA, USA; 4 International Centre for Equity in Health, Post-Graduate Program in Epidemiology, Federal University of Pelotas, Brazil

## Abstract

**Background:**

Expanding social protection programme is a major target of the Sustainable Development Goals. Previous studies provided evidence for the relationship of social protection programme to greater use of health services and some improved health outcomes for children. Yet, its impact on child mortality has not been clearly revealed. In this study, we examined the association between social protection programmes and child mortality.

**Methods:**

We obtained child mortality data from 379 nationally representative surveys involving 101 low- and middle-income countries (LMICs). We included five child mortality outcomes in the study, which were neonatal mortality rate (NMR), post-neonatal mortality rate (PMR), childhood mortality rate (CMR), infant mortality rate (IMR), and under-5 mortality rate (U5MR). We extracted data on social protection programmes from multiple data sources (eg, Atlas of Social Protection Indicators of Resilience and Equity). Social protection and labour programme (SPL) was the major type of social protection we included. We also included four subtypes of SPL - social assistance, cash transfer, social insurance, and labour market protection. Both unadjusted and adjusted regressions were conducted to measure the associations between characteristics of social protection programmes and child mortality, as well as inequalities in child mortality.

**Results:**

Among the 101 countries, the median coverage rate of SPL was 28.5%, with an interquartile range between 6.5% and 55.2%. Using the adjusted model, we found a one-percentage-point increase in SPL coverage is associated with a reduction of 0.09 (95% confidence interval (CI) = 0.04, 0.14) per 1000 live births in NMR, 0.11 (95% CI = 0.04, 0.18) in PMR, and 0.25 (95% CI = 0.11, 0.38) in CMR. Social assistance programme was the only subtype of SPL to be significantly associated with lower mortality rates. A higher SPL coverage was associated with better equity in child mortality – as the coverage of SPL increased by one percentage point, the concentration index of CMR would increase by 0.08 (95% CI = 0.03, 0.13) in the adjusted model, suggesting an improvement in equity.

**Conclusions:**

The strong association between social protection programme and child mortality suggests that to achieve the SDG targets of universal social protection and to reduce child mortality, LMICs shall consider prioritizing the expansion of social protection programmes.

Social protection programmes, particularly social protection floors, play a crucial role in addressing poverty, food security, and access to education and health [1] Social protection has long been proposed and used as a means for industrialized countries to protect their most vulnerable citizens [2] As the Sustainable Development Goals (SDG) era came into sight in 2015, implementing and expanding nationally-appropriate social protection systems has been, for the first time, prioritized in the global agenda for both high-income and low- and middle-income countries (LMICs) [3] Although some countries, such as Argentina, Brazil, Mongolia, have achieved or almost achieved universal coverage of social protection, a number of LMICs are still struggling with limited coverage, inadequate benefit levels, and disproportionate distribution of the benefits [1]. In low-income countries, only 1 in 5 of the poorest are covered by any type of social protection programmes (eg, cash, in-kind transfers, social pensions, etc.) [4].

Despite their importance, social protection programmes have not been clearly defined. During the past decade, social protection is increasingly recognized as a set of public actions that address poverty, vulnerability, and risk throughout the lifecycle. Previous literature has divided social protection into various subtypes, including social assistance, social insurance, and labour market protection [5]. Social assistance refers to the non-contributory interventions with the fee paid by the providers or government; cash transfer [CT] is an example [6]. Social insurance is usually in a contributory form, which requires regular payments from the participants, although sometimes subsidized by the government or other organizations. Labour market interventions include unemployment benefits, active protection to improve labour market participation, and passive protection to ensure minimum employment standards (eg, maternity benefits) [6].

Recent studies provided evidence for the impact of social protection programmes on population health. One well recognized area is tuberculosis (TB) prevention and treatment – previous studies found that the higher coverage of social protection programmes was strongly associated with lower global incidence of TB; social insurance appeared to be more correlated with TB incidence comparing to social assistance and labour protection [7,8]. As to child and maternal health, it was documented that social protection programmes were linked with lower cost-related barriers to health services, and could therefore, increase the use of preventive services, immunization coverage, and perinatal care [9-11]. The most widely studied social protection programme is CT. Multiple studies have revealed the effects of CT programmes on health service utilization, particularly prenatal care, delivery care, and child vaccination services, and certain health outcomes, such as nutritional status, cognitive outcomes, and morbidity [11-21]. However, the evidence for the impact of CT programmes on mortality remained scarce and inconsistent [22-28].

In the SDG era, ambitious targets have been set to reduce neonatal mortality to 12 deaths per 1000 live births and under-5 mortality to 25 deaths per 1000 live births by 2030. Child mortality has a complex interaction with poverty and its related social and structural factors [29-31]. Social protection programme, with the major target to protect the disadvantaged population from vulnerability, serves as an essential part in the poverty reduction actions [7], yet its impact on child mortality has not been systematically analysed. Moreover, despite designed to support the disadvantaged population, whether social protection has managed to provide a stronger support for the poorer children needs to be carefully evaluated. In this study, our aim was to investigate the association between social protection programmes (ie, social assistance, social insurance, and labour market protection) and the level and equity status of child mortality.

## METHODS

### Data sources

The mortality data was derived from the nationally representative Demographic and Health Survey (DHS), Multiple Indicator Cluster Survey (MICS), and Reproductive Health Survey (RHS). We involved 379 surveys conducted between 1991 and 2019 in 101 LMICs (Table S1 in the **Online Supplementary Document**). On average, each country has conducted 3.7 rounds of surveys. The median of the latest survey year is 2016, with an interquartile range between 2014 and 2018. DHS, MICS, and RHS are comparable data sets [32,33], which all use extensive interviewer training, standardized measurement tools and techniques, identical core questionnaire, and instrument pretesting to ensure standardization and comparability across diverse sites and time [34,35]. DHS, MICS, and RHS also provide established wealth index and wealth quintiles based on the ownership of selected assets, such as televisions, bicycles, types of water access, etc [36].

Data on social protection programmes were extracted from four main data sources, including the Atlas of Social Protection Indicators of Resilience and Equity (ASPIRE) of the World Bank, SDG data repository, the database of International Labour Organization, and the series of reports on social policies conducted by the United Nations [3,37-39]. We obtained coverage and benefit levels of social assistance, CT, social insurance, and labour market programmes.

We also drew data on GDP per capita, number of physicians per 1000 people, number of nurses per 1000 people, and population size from the World Bank data set [40]. The GDP per capita used in the analysis was obtained from the World Bank; we converted it using purchasing power parity to constant 2011 US$ [41,42]. We obtained the variables of current health expenditure as a percentage of GDP and public health expenditure as a percentage of GDP from the WHO Global Health Expenditure Data [43]. We derived the prevalence of mothers not finishing primary school from the DHS data sets.

### Health outcomes

Our main outcome was child mortality. We included five indicators in our study, which were neonatal mortality rate (NMR), post-neonatal mortality rate (PMR), childhood mortality rate (CMR), infant mortality rate (IMR), and under-5 mortality rate (U5MR). NMR, IMR, and U5MR are the most commonly used indicators to measure child mortality; they referred to the number of deaths occurred in the first month, the first year, and the first five years of life per 1000 live births, respectively [44]. In this study, we also included PMR and CMR. PMR and CMR referred to the number of deaths occurred between 1 month and 1 year old, and between 1 and 5 years old per 1000 live births, respectively [44]. Complete definitions of mortality rates were listed in **Table 1**.

**Table 1:** Definition and reference of the variables

Variable	Definition	Reference
Beneficiary incidence in the poorest quintile (Q1)	The sum of all benefits received by individuals in Q1 as a proportion of the sum of all benefits received by all individuals in the population. For example, the beneficiary incidence of social protection and labour programme in Q1 referred to the sum of benefits of all related policies and programmes in Q1 as a proportion of the overall benefits received by the general population	[37]
Cash transfer	Direct cash transfer given to children/households	[6,38]
Childhood mortality rate	The probability of a child exposed in a specific period dying on or after their first birthday but before reaching the age of five years	[44]
Coverage of social protection programme in general	The proportion of the individuals covered by the programme in total population. For example, the coverage of SPL was defined as the proportion of population covered by one or more types of social protection and labour (SPL) programmes; the coverage of a sub-type programme (eg, social assistance) was defined as the proportion of the population covered by that certain type of programme	[37]
Coverage of social protection programme in Q1	The proportion of the individuals covered by the programme in Q1 group. For example, the coverage of SPL in Q1 was defined as the proportion of population in Q1 group covered by one or more types of SPL programmes; the coverage of a sub-type programme (eg, social assistance) in Q1 was defined as the proportion of the population in Q1 group covered by that certain type of programme	[37]
Infant mortality rate	The probability of a child exposed in a specific period dying before reaching their first birthday, expressed as a rate per 1000 live births	[44]
Input per beneficiary in general	The sum of benefits received by the programme beneficiaries divided by the programme beneficiaries. For example, the input per beneficiary of SPL was defined as the sum of all benefits from related policies and programmes divided by the population size who received one or more types of SPL	[37]
Input per beneficiary in Q1	The sum of benefits received by the programme beneficiaries in Q1 group divided by the programme beneficiaries in Q1	[37]
Labour market protection	Unemployment benefits and active (promoting labour market participation) or passive (ensuring minimum employment standards)	[6]
Neonatal mortality rate	The probability of a child exposed in a specific period dying before reaching the age of 1 mo, expressed as a rate per 1000 live births	[44]
Post-neonatal mortality rate	The probability of a child exposed in a specific period on or after the age of 1 mo but before reaching the age of 1 y, calculated as the difference between the infant mortality rate and the neonatal mortality rate, expressed as a rate per 1000 live births	[44]
Social assistance	Non-contributory transfers in cash, vouchers, or in-kind (including school feeding) to individuals or households in need; public works programmes; fee waivers (for basic health and education services); and subsidies (eg, for food, fuel)	[6,37]
Social insurance	Contributory and beneficiaries receive benefits or services in recognition of contributions to an insurance scheme	[37]
Social protection and labour programme	The set of policies and programmes designed to reduce poverty and vulnerability, including social assistance programmes, social insurance programmes, and labour market protection	[45,46]
Under-5 mortality rate	The probability (expressed as a rate per 1000 live births) of a child exposed in a specific period dying before reaching the age of five years, expressed as a rate per 1000 live births	[44]

### Exposures

Social protection programmes were the major exposures. In this study, we examined social protection and labour (SPL) programme in general, which included all policies and programmes designed to reduce poverty and vulnerability. We also investigated four sub-types of SPL programmes, including social assistance, CT, social insurance, and labour market protection. Though CT is a sub-type of social assistance, we chose to include it separately to make our results more comparable with previous research, since CT is the most widely studied social protection programme. Detailed definition of social protection programmes was listed in **Table 1**.

In this paper, we mainly focused on the coverage of social protection programmes. We included two coverage indicators - the coverage of the social protection programme in general and the coverage of the social protection programme in the poorest quintile (Q1). The coverage of the social protection programme referred to the proportion of the individuals covered by the programme in total population. The coverage of the social protection programme in the poorest quintile (Q1) referred to the proportion of the individuals covered by the programme in Q1 group. Besides the coverage of social protection programmes, we also examined how beneficiary incidence, input per beneficiary in general, and input per beneficiary in Q1 of social protection programmes affected child mortality. See detailed definition in **Table 1**.

### Inequality measurement

Following previous studies [29,47], we adopted three most commonly used inequality measurements, including concentration index (CIX), slope index of inequality (SII), and the difference between the richest quintile Q5 and Q1.

CIX was generated from the concentration curves that plot the cumulative proportion of one variable against the cumulative proportion of the population ranked by wealth. CIX captured the extent to which health outcomes/ health interventions differ across individuals’ ranks by wealth [48] CIX was expressed on a scale ranging from −100 to 100, with zero representing perfect equality; negative values meant that mortality was more prevalent among the poor, while positive values represent the indicator was more prevalent in the rich.

SII measured absolute inequality and represented the absolute difference in percentage points between the fitted coverage levels at the extremes of the wealth distribution, through a linear regression [48]. SII scaled from −100 to +100, with zero indicating perfect equality; negative values meant that the outcome was more prevalent in the poor, while positive values meant that the indicator was more prevalent in the rich.

Both CIX and SII took the entire distribution of child mortality over the wealth indexes into account. Yet, their values were not straightforward to understand. Therefore, we also generated the difference in the mortality rate between the Q5 and Q1 groups. Positive values suggested the mortality rate to be higher among the Q5 than Q1, while negative values suggested the mortality rate to be higher among the Q1 than Q5. Zero represented perfect equality.

### Statistical analysis

We assessed the association between the characteristics of social protection programmes and mortality rate using ordinary least squares (OLS) regressions. We developed two sets of OLS regression models for each outcome and exposure variable to test the consistency of the results.

First, we ran unadjusted regressions using the most recent surveys from each country. Second, we ran adjusted models controlling for GDP per capita, density of health professionals, health expenditure variables, and population size in the regression. We performed similar regressions to examine the associations between social protection programmes and inequalities in mortality rates.

Social protection indicators and the density of health professionals had fairly large (>20%) amounts of missing data (Table S2 in the **Online Supplementary Document**). The proportion of missingness was similar across countries from various regions and with different income levels, suggesting the missingness was likely to be random [7,49]. To handle the missing data on these indicators, we followed previous studies[7,48] and performed multiple imputation of these items, along with poverty headcount ratio at US$1.9 a day, employment to population ratio, and annual GDP growth, using the STATA command of MI. We presented comparison of the observed and imputed data with histograms in Figure S1 in the **Online Supplementary Document**. The distribution of the observed and the imputed data were highly comparable.

We used STATA (version 14.2) (StataCorp, College Station, TX, USA) for all analyses procedures. All statistical tests were 2-tailed, and *P* < 0.05 was considered statistically significant.

### Sensitivity analysis

To control for other country-specific characteristics that cannot be fully addressed in the adjusted model above, we conducted sensitivity analysis by running country-fixed effect model using all available surveys. In this model, besides controlling for all the covariates as in the adjusted model above, we added country-fixed effect to control for unobservable country-specific characteristics (eg, religion, race, culture, etc.) that were invariant over time.

## RESULTS

### Summary of the sample characteristics

We summarized the key characteristics of the sample in **Table 2**. Among the 101 countries involved in the study, the median of the NMR was 24.1 per 1000 live births (interquartile range (IQR) = 14.9, 29.7); the median of PMR was 16.7 per 1000 live births (IQR = 8.2, 23.4); the median of CMR was 13.4 per 1000 live births (IQR = 4.5, 26.3).

**Table 2:** Summary table of key characteristics, the most recent survey years

	Median (IQR)	Lowest value* (95% CI if available)	Highest value† (95% CI if available)
***Health outcome indicators***			
Neonatal mortality rate per 1000 live births (NMR)	24.1 (14.9, 29.7)	3.4 (1.4, 5.4)	44.3 (39.4, 49.3)
Post-neonatal mortality rate per 1000 live births (PMR)	16.7 (8.2, 23.4)	1.8 (0.1, 3.4)	48.3 (43.2, 53.4)
Childhood mortality rate per 1000 live births (CMR)	13.4 (4.5, 26.3)	0.6 (-0.2, 1.5)	94.3 (88.3, 100.3)
Infant mortality rate per 1000 live births (IMR)	41.4 (23.7, 52.7)	5.1 (2.6, 7.7)	85.0 (77.6, 92.4)
Under-5 mortality rate per 1000 live births (U5MR)	54.2 (29.5, 77.7)	5.8 (3.1, 8.5)	153.0 (145.3, 160.7)
***Inequalities in health outcome indicators***			
**Absolute difference between Q5 and Q1 (deaths per 1000 live births):**			
NMR	-7.4 (-11.9, -1.9)	-26.2 (-29.5, -22.9)	12.7 (6.4, 19.0)
PMR	-10.5 (-15.1, -4.5)	-37.5 (-42.5, -32.5)	8.6 (4.0, 13.2)
CMR	-11.7 (-20.9, -4.4)	-88.4 (-94.9, -81.9)	1.8 (-2.7, 6.3)
IMR	-16.9 (-24.3, -9.3)	-55.4 (-60.5, -50.3)	12.9 (9.8, 16.0)
U5MR	-26.2 (-40.6, -15.6)	-119.4 (-125.9, -112.9)	14.5 (10.8, 18.2)
**Concentration index:**			
NMR	-6.7 (-12.5, -0.1)	-30.0 (-53.5, -6.4)	16.7 (5.9, 27.5)
PMR	-13.5 (-18.6, -7.3)	-71.8 (-104.1, -39.4)	10.3 (-7.6, 28.1)
CMR	-17.4 (-25.8, -11.9)	-62.9 (-90.5, -35.4)	10.2 (-20.1, 40.5)
IMR	-9.6 (-14.5, -4.0)	-44.4 (-64.7, -24.0)	11.9 (2.2, 21.7)
U5MR	-11.8 (-16.3, -6.1)	-46.4 (-65.8, -26.9)	7.7 (-0.7, 16.0)
**Slope index of inequality (deaths per 1000 live births):**			
NMR	-8.1(-14.3, -0.2)	-32.3 (-35.9, -28.7)	13.2 (6.3, 20.0)
PMR	-11.3 (-18.3, -5.3)	-46.7 (-49.1, -44.4)	7.2 (-1.2, 15.5)
CMR	-13.4 (-23.1, -4.8)	-116.7 (-150.3, -83.0)	2.7 (-2.7, 8.0)
IMR	-19.8 (-29.2, -9.8)	-72.0 (-95.2, -48.8)	13.7 (3.9, 23.5)
U5MR	-30.5 (-46.8, -16.8)	-153.6 (-203.8, -103.4)	13.7 (4.7, 22.6)
***Social protection characteristics***			
**Social protection coverage (%):**			
**All social protection and labour programmes**	28.5 (6.5, 55.2)	1.3	99.9
Social assistance	16.7 (7.4, 44.9)	0.6	99.8
Cash transfer	2.6 (1.1, 8.4)	0.0	99.8
Social insurance	6.0 (1.9, 21.3)	0.4	59.5
Labour market protection	3.3 (1.2, 5.7)	0.1	25.8
**Social protection coverage in Q1 (%):**			
**All social protection and labour programmes**	35.0 (6.6, 63.7)	0.0	99.8
Social assistance	26.1 (12.5, 54.9)	0.0	99.8
Cash transfer	1.2 (0.0, 9.9)	0.0	99.8
Social insurance	1.2 (0.5, 20.1)	0.0	60.6
Labour market protection	2.7 (0.8, 4.7)	0.1	25.3
**Beneficiary incidence in the poorest quintile (Q1) (%):**			
**All social protection and labour programmes**	22.1 (17.8, 25.3)	0.0	35.2
Social assistance	24.8 (21.0, 28.3)	0.0	53.3
Cash transfer	30.1 (21.1, 35.8)	5.4	70.6
Social insurance	8.0 (3.7, 20.3)	0.0	32.0
Labour market protection	18.5 (11.1, 23.9)	2.9	38.4
**Social protection input per beneficiary (US dollars):**			
**All social protection and labour programmes**	0.48 (0.23, 1.52)	0.00	38.15
Social assistance	0.19 (0.08, 0.41)	0.00	17.90
Cash transfer	0.40 (0.18, 0.92)	0.00	8.90
Social insurance	1.39 (0.76, 2.41)	0.01	50.77
Labour market protection	0.17 (0.06, 0.25)	0.02	1.40
**Social protection input per beneficiary in Q1 (US$):**			
**All social protection and labour programmes**	0.13 (0.03, 0.40)	0.00	42.52
Social assistance	0.13 (0.04, 0.34)	0.00	14.61
Cash transfer	0.39 (0.12, 0.76)	0.00	8.22
Social insurance	0.43 (0.17, 1.26)	0.00	93.19
Labour market protection	0.08 (0.04, 0.16)	0.00	0.70

NMR – neonatal mortality rate, PMR – post-neonatal mortality rate, CMR – childhood mortality rate, IMR – infant mortality rate, U5MR – under-5 mortality rate, CI – confidence interval

*“Lowest value” is the lowest value of the specific indicator among all countries.

†“Highest value” is the highest value of the specific indicator among all countries.

Child deaths were more prevalent among the poorer population in most countries; the extent varied largely across countries. In the vast majority of countries, the differences between Q5 and Q1 groups in mortality rates were negative, suggesting that deaths were more prevalent in the poorest population than in the richest population. For example, the absolute difference between Q5 and Q1 in NMR was with a median of -7.4 per 1000 live births (IQR = -11.9, -1.9); it ranged widely from -26.2 (95% confidence interval (CI) = -29.5, -22.9) in India in 2015 to 12.7 (95% CI = 6.4, 19.0) in Maldives in 2016. Other mortality indicators and inequality measurements showed similar trends.

The coverage and benefit levels of social protection programmes were generally low in most of the countries, with large cross-country variations. Among the 101 countries, the most recent data showed that the median proportion of the population covered by SPL was 28.5% (IQR = 6.5%, 55.2%), ranging from less than 5% in Mali, Comoros and Zambia, to almost universal coverage in Mongolia. The coverage of SPL in Q1 was slightly higher than in the general population, with a median of 35.0% (IQR = 6.6, 63.7). The beneficiary incidence showed that a median of 22.1% (IQR = 17.8, 25.3) of the benefits of SPL were received by the Q1 group. The input per beneficiary of SPL was low in most countries; the median value was US$0.48 (IQR = 0.23, 1.52) for the general population, and US$0.13 (IQR = 0.03, 0.40) for the Q1 group.

### Social protection programmes and child mortality rates

**Figure 1:**
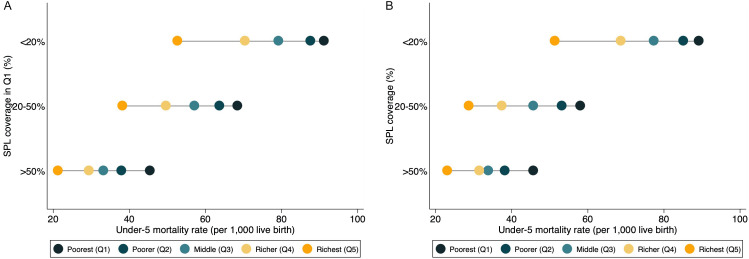
The relationship between the coverage of social protection and labour (SPL) programme and under-5 mortality rate by quintile, using data from the most recent survey years. **Panel A. **Coverage of SPL (%) and under-5 mortality rate. **Panel B.** Coverage of SPL in Q1 (%) and under-5 mortality rate.

We generated intuitive graphs to show the relationship between the characteristics of social protection programmes and mortality indicators using data from the most recent years in **Figure 1** and Figures S2 and S3 in the **Online Supplementary Document**. In most cases, the better-off population had lower mortality rate. For example, in countries with the less than 20% of the population covered by SPL, U5MR in Q5 group was on average 89.1 per 1000 live births, comparing to 51.3 per 1000 live births in Q1 group.

The unadjusted and adjusted associations between the coverage of social protection programmes and mortality indicators can be found in **Table 3**. In the unadjusted model, we found that the coverage of SPL in general and in Q1 were significantly associated with lower mortality rates in all periods of child life. For example, a one percentage point increase in the coverage of SPL in Q1 was significantly associated with a reduction of 0.18 (95% CI = 0.13, 0.23) per 1000 live births in NMR, 0.17 (95% CI = 0.12, 0.22) in PMR, and 0.33 (95% CI = 0.24, 0.42) in CMR. Although most of the coefficients attenuated after covariate adjustment, the results remained significant. For example, the association between the coverage of SPL in Q1 and CMR reduced to 0.24 (95% CI = 0.13, 0.35) per 1000 live births, but was still significant at 0.001 significant level (**Table 3**).

**Table 3:** Association between the coverage of social protection and labour programmes and mortality indicators^*^

	All social protection and labour programmes	
	**Unadjusted**	**Adjusted**
**Social protection coverage (%):**		
NMR	-0.20† (-0.25, -0.14)	-0.09† (-0.14, -0.04)
PMR	-0.20† (-0.25, -0.14)	-0.11‡ (-0.18, -0.04)
CMR	-0.37† (-0.48, -0.26)	-0.25† (-0.38, -0.11)
IMR	-0.40† (-0.50, -0.30)	-0.19† (-0.29, -0.10)
U5MR	-0.74† (-0.92, -0.56)	-0.43† (-0.64, -0.23)
**Social protection coverage in Q1 (%):**		
NMR	-0.18† (-0.23, -0.13)	-0.10† (-0.16, -0.04)
PMR	-0.17† (-0.22, -0.12)	-0.09‡ (-0.15, -0.03)
CMR	-0.33† (-0.42, -0.24)	-0.24† (-0.35, -0.13)
IMR	-0.35† (-0.43, -0.26)	-0.18† (-0.27, -0.09)
U5MR	-0.66† (-0.81, -0.50)	-0.39† (-0.56, -0.22)

NMR – neonatal mortality rate, PMR – post-neonatal mortality rate, CMR – childhood mortality rate, IMR – infant mortality rate, U5MR – under-5 mortality rate

*In the adjusted model, we controlled for GDP per capita, current health expenditure as a percentage of GDP, public health expenditure as a percentage of GDP, physician density per 1000 people, nurse density per 1000 people, population size, and the prevalence of mothers not finishing primary school.

†*P*<0.001.

‡*P*<0.01

The results on beneficiary incidence and input per beneficiary of SPL were presented in Table S3 in the **Online Supplementary Document**. Higher beneficiary incidence in Q1 appeared to be significantly associated with lower PMR, CMR, and U5MR in both the unadjusted and adjusted models. We did not find significant associations between the input per beneficiary of SPL and child mortality rates either in general or in Q1 group.

We showed the results for the subtypes of SPL and child mortality in Table S4 in the **Online Supplementary Document****.** Higher coverage of social assistance programme in general was significantly associated with lower mortality rates in all periods of child life. For example, we found that a one percentage point increase in the coverage of social assistance programme was associated with a decrease of 0.38 (95% CI = 0.27, 0.50) per 1000 live births in U5MR in the adjusted model. The coverage of social assistance programme in Q1 showed similar trends. Furthermore, the beneficiary incidence of social assistance in Q1 was also associated with lower mortality rates in all periods of child life. For example, as the beneficiary incidence of social assistance in Q1 increased by one percentage point, the U5MR would reduce by 0.97 (95% CI = 0.57, 1.37) in the adjusted model. In contrast, the coverage or benefits levels of other subtypes of SPL (ie, CT, social insurance, labour market protection) were insignificantly or only weakly associated with child mortality.

### Social protection programmes and inequalities in child mortality

The association between the characteristics of social protection programmes and equity status of child mortality was presented in **Table 4** and Tables S5-7 in the **Online Supplementary Document**. The unadjusted results showed that higher coverage of SPL in general and in Q1 was both significantly associated with better equity status of PMR, CMR and U5MR. After covariate adjustment, the magnitudes of most associations attenuated and became insignificant; however, higher coverage of SPL in general or in Q1 were still significantly associated with better equity of CMR. For example, as the coverage of SPL increased by one percentage point, the CIX of CMR would increase significantly by 0.08 (95% CI = 0.03, 0.13) in the adjusted model, indicating an improvement in equity (**Table 4**).

**Table 4:** Association between social protection programmes and concentration index of mortality indicators*

	All social protection and labour programmes			
	**Unadjusted**		**Adjusted**	
	**Coefficient**	**Reducing inequalities**	**Coefficient**	**Reducing inequalities**
**Social protection coverage (%):**				
NMR	0.09 (-0.01, 0.19)	No	0.03 (-0.08, 0.13)	No
PMR	0.13 (0.05, 0.21)	Yes	0.03 (-0.05, 0.12)	No
CMR	0.10 (0.05, 0.16)	Yes	0.08 (0.03, 0.13)	Yes
IMR	0.07 (-0.02, 0.16)	No	0.03 (-0.02, 0.08)	No
U5MR	0.09 (0.03, 0.14)	Yes	0.04 (-0.03, 0.11)	No
**Social protection coverage in Q1 (%):**				
NMR	0.08 (-0.01, 0.16)	No	0.02 (-0.06, 0.10)	No
PMR	0.12 (0.05, 0.18)	Yes	0.04 (-0.03, 0.11)	No
CMR	0.10 (0.05, 0.15)	Yes	0.07 (0.04, 0.10)	Yes
IMR	0.09 (0.05, 0.14)	Yes	0.05 (-0.00, 0.10)	No
U5MR	0.08 (0.03, 0.13)	Yes	0.04 (-0.01, 0.09)	No

NMR – neonatal mortality rate, PMR – post-neonatal mortality rate, CMR – childhood mortality rate, IMR – infant mortality rate, U5MR – under-5 mortality rate, No – no significant change in equity status

*In the adjusted model, we controlled for GDP per capita, current health expenditure as a percentage of GDP, public health expenditure as a percentage of GDP, physician density per 1000 people, nurse density per 1000 people, population size, and the prevalence of mothers not finishing primary school.

Table S5 in the **Online Supplementary Document** showed the results on beneficiary incidence and input per beneficiary. Beneficiary incidence of SPL in Q1 appeared to be significantly associated with CIX in the post-neonatal and childhood periods, but not in the neonatal period. For example, as the beneficiary incidence of SPL increased by one percentage point, the CIX of CMR would significantly increase by 0.27 (95% CI: 0.04, 0.51) in the adjusted model. We did not find the per beneficiary input of SPL to be associated with CIX in any period of child life.

We measured inequality with SII and difference between Q5 and Q1 groups in Tables S6 and S7 in the **Online Supplementary Document**. The results were consistent with above.

### Sensitivity analysis

In the sensitivity analysis, we used all available surveys in the regression. We controlled for country-fixed effect and covariates involved in the adjusted model as above. The results were presented in Tables S8 to S12 in the **Online Supplementary Document**. The results from sensitivity analysis were very similar as the ones from the adjusted model. For example, the association between coverage of SPL in Q1 and U5MR was 0.39 (95% CI = 0.22, 0.57) in the adjusted model and 0.35 (95% CI = 0.20, 0.49) in the sensitivity analysis (Table S8 in the **Online Supplementary Document**).

## DISCUSSION

Four salient findings emerged from our analysis using data from 101 LMICs. First, the coverage of SPL, both in general and in Q1, was significantly associated with lower mortality rates in all periods of child life. Second, when investigating the subtypes of SPL, the coverage of social assistance programmes was more associated with child mortality comparing to the other subtypes of SPL. Third, a higher coverage of SPL was associated with better equity status in the childhood period, but not in earlier periods of life. Last, input per beneficiary did not show any apparent association with either child mortality or inequality in child mortality.

Our result on the strong association between the coverage of social protection programmes and child mortality provided evidence to the SDG target of moving towards universal social protection. It also shed light on a long-existing dilemma that social programme designers faced, particularly in LMICs - given the scarcity of resources, whether the policymakers should expand the coverage of social protection programmes or provide higher benefit levels to the most disadvantaged population [50,51]. This dilemma was related to the debate on universal approach vs targeting approach that has been ongoing for decades [5,51]. Our study provided clear evidence embracing a higher coverage of social protection programmes. However, we did not intend to downplay the role of the targeting programmes. Actually, we support a mixture of universal and targeted approaches – since a higher coverage of social protection programmes in Q1 was strongly associated with better health outcomes, the policymakers might first expand these programmes among the most disadvantaged population to break the vicious circle between poverty and worse health [5,7,51]; as a next step, the policymakers could gradually expand these programmes to more affluent population; when health resources became more affluent, the policymakers could further improve benefit levels of the population. Yet notably, as countries varied substantially regarding their culture, politics, health resources, etc., detailed discussion should be based on a country-specific context.

We also found that compared with CT, social assistance was more associated with mortality rate. Previous studies mainly focused on the impact of CT on neonatal mortality, with little evidence for other types of mortality rates and the impact of social assistance [22-28]. Studies investigating CT in Brazil, Mexico, India, Nigeria, and Nepal found no association between CT and neonatal mortality, which is consistent with our study [23-28]. Studies on infant mortality, post-neonatal mortality, and under-5 mortality have been very scarce – two studies in Brazil and Mexico found that CT was associated with a reduction in infant mortality [24,28]; two studies in Brazil and Nigeria showed inconsistent results for post-neonatal mortality [25,28]; only one study included under-5 mortality as outcomes, and found significant association [22]. Our findings provided new evidence, showing CT was not significantly associated with infant mortality, post-neonatal mortality, and under-5 mortality. Yet, notably, we also found social assistance was associated with all the above mortality rates. Though CT is an important part of social assistance, the latter also include other types of social protection such as fee waivers and subsidies, which may contribute to the difference in effects on mortality between CT and social assistance. The relationship between CT programmes and child protection efforts is complex. Despite the well-documented short-term impacts of CT programme (reduce monetary poverty, improve access to health services, etc.), CT programmes, especially unconditional cash transfers, has been long criticized for triggering expenditure on temptation goods (alcohol and cigarette consumption), reducing labour force participation, and missing the targeted population [9] Consequently, the money transferred via CT programmes might not necessarily end up on the intended population or the intended aspects. Conditional cash transfer (CCT) could, to some degree, alleviate this problem [28] Yet in this study, we did not have valid data to examine the effects of CCT.

Social protection programmes were also found to be significantly associated with equity status of childhood mortality, but not neonatal mortality. The difference in the effects by periods of child life could be explained by the various interventions needed to address deaths in each period. The three major causes of childhood deaths were pneumonia, diarrhoea, and malaria in LMICs, for which there are many effective household and community interventions that can be improved by social protection programmes [52] While the underlying solutions of neonatal deaths (eg, preterm births, severe infections, asphyxia) largely lie on high-quality facility-based and outreach services [53] Those system-based interventions were much harder to be carried out than the household/community-based interventions; which might be a critical reason for the difficulty to alleviate earlier-stage deaths among the disadvantaged population.

There are some limitations to this study. First, the coverage and benefit levels of social protection programmes had substantial amount of missing values. To deal with this issue, we followed previous studies [7,54] and adopted a robust protocol to estimate the missing data; we also did comparison between the observed and imputed data to ensure the validity of our estimation. Yet the analysis could be refined with improved data availability and quality [7,49]. Second, the observational data and cross-sectional analysis limit our capacity to make any causal inference. Third, the classification of the poorest quintile adopted in this study is country-specific and time-sensitive. The poorest quintile in an upper-middle-income country could be better off than the richer quintiles in a less developed country, which might have an impact on the cross-national comparison [29]. Fourth, we were unable to include HIV prevalence for each country as a covariate due to the high volume of missingness, which is a common risk factor of child mortality [55]. Last, our data cannot support separate analyses of conditional and unconditional cash transfer.

## CONCLUSIONS

Despite the limitations, this is the first known study systematically examining the relationship between social protection programmes and child mortality, covering countries in Africa, Asia, Latin America, and Oceania. Moving towards the SDG targets of universal social protection and child mortality, we suggested to expand the coverage of social protection programmes based on a country-specific discussion. CT, although embraced in many developing countries with a trend of universal provision, need to be further analysed. To mitigate inequalities in child health, particularly in the infant period, we suggest a more comprehensive set of supporting policies (eg, high-quality health services, well-informed caregivers, social norms affecting health behaviours, etc.) to allow sustainable improvements on child health.

## Additional material

Online Supplementary Document
