# Supplementary material for: Social protection and the level and inequality of child mortality in 101 low- and middle-income countries: A statistical modelling analysis
Source: J Glob Health. 2021 Oct 23;11:04067. doi: 10.7189/jogh.11.04067 (PMC8561336; doi:10.7189/jogh.11.04067)
Supplement: Online Supplementary Document [file jogh-11-04067-s001.pdf]

Table S1. List of countries, years, and data sources included in the study

Country	Year	Source[1]
Afghanistan	2010, 2015	MICS, DHS
Albania	2002, 2005, 2008, 2017	RHS, MICS, DHS
Angola	2015	DHS
Argentina	2011	MICS
Armenia	2000, 2005, 2010, 2015	DHS
Azerbaijan	2006	DHS
Bangladesh	1993, 1996, 1999, 2004, 2006, 2007, 2011, 2012, 2014, 2019	DHS, MICS
Belarus	2005, 2012	MICS
Belize	1991, 2006, 2011, 2015	RHS, MICS
Benin	1996, 2001, 2006, 2011, 2014, 2017	DHS, MICS
Bhutan	2010	MICS
Bolivia	1994, 1998, 2003, 2008	DHS
Bosnia and Herzegovina	2006, 2011	MICS
Brazil	1996, 2006, 2013	DHS, NSS
Burkina Faso	1998, 2003, 2006, 2010	DHS, MICS
Burundi	2005, 2010, 2016	DHS, MICS
Cambodia	2000, 2005, 2010, 2014	DHS
Cameroon	1998, 2004, 2006, 2011, 2014, 2018	DHS, MICS
Central African Republic	1994, 2006, 2010	DHS, MICS
Chad	1996, 2004, 2010, 2014	DHS, MICS
Colombia	1995, 2000, 2005, 2010, 2015	DHS
Comoros	1996, 2012	DHS
Congo	2005, 2011, 2014	DHS, MICS
Costa Rica	1992, 2011	RHS, MICS
CÙte d'Ivoire	1994, 1998, 2006, 2011, 2016	DHS, MICS
Democratic Republic of the Congo	2007, 2010, 2013, 2017	DHS, MICS
Dominican Republic	1996, 1999, 2002, 2007, 2013, 2014	DHS, MICS
Ecuador	1994, 1999, 2004, 2012	RHS, NSS
Egypt	1995, 2000, 2005, 2008, 2014	DHS
El Salvador	1993, 1998, 2002, 2008, 2014	RHS, MICS
Eswatini	2006, 2010, 2014	DHS, MICS
Ethiopia	2000, 2005, 2011, 2016	DHS
Gabon	2000, 2012	DHS
Gambia	2005, 2010, 2013, 2018	DHS, MICS
Georgia	2005, 2018	MICS
Ghana	1993, 1998, 2003, 2006, 2008, 2011, 2014, 2017	DHS, MICS

Guatemala	1995, 1998, 2002, 2008, 2014	RHS, DHS
Guinea	1999, 2005, 2012, 2016, 2018	DHS, MICS
Guinea-Bissau	2006, 2014	MICS
Guyana	2006, 2009, 2014	DHS, MICS
Haiti	1994, 2000, 2005, 2012, 2016	DHS
Honduras	1991, 1996, 2001, 2005, 2011	DHS, RHS
India	1998, 2005, 2015	DHS
Indonesia	1997, 2002, 2007, 2012, 2017	DHS
Iraq	2011, 2018	MICS
Jamaica	2011	MICS
Jordan	1997, 2002, 2007, 2012, 2017	DHS
Kazakhstan	1995, 1999, 2006, 2010, 2015	DHS, MICS
Kenya	1993, 1998, 2003, 2008, 2014	DHS
Kyrgyzstan	1997, 2005, 2012, 2014, 2018	DHS, MICS
Lesotho	2004, 2009, 2014, 2018	DHS, MICS
Liberia	2007, 2013	DHS
Madagascar	1997, 2003, 2008, 2018	DHS, MICS
Malawi	2000, 2004, 2006, 2010, 2013, 2015	DHS, MICS
Maldives	2009, 2016	DHS
Mali	1995, 2001, 2006, 2009, 2012, 2015, 2018	DHS, MICS
Mauritania	2007, 2011, 2015	MICS
Mexico	2015	MICS
Mongolia	2005, 2010, 2013, 2018	MICS
Montenegro	2005, 2013, 2018	MICS
Morocco	2003	DHS
Mozambique	1997, 2003, 2008, 2011, 2015	DHS, MICS
Myanmar	2015	DHS
Namibia	2000, 2006, 2013	DHS
Nepal	1996, 2001, 2006, 2010, 2011, 2014, 2016	DHS, MICS
Nicaragua	1992, 1997, 2001, 2006	RHS, DHS
Niger	1998, 2006, 2012	DHS
Nigeria	2003, 2007, 2008, 2011, 2013, 2016, 2018	DHS, MICS
North Macedonia	2005, 2011	MICS
Pakistan	2006, 2012, 2017	DHS
Panama	2013	MICS
Paraguay	1995, 1998, 2004, 2008, 2016	RHS, MICS
Peru	1996, 2000, 2004, 2005, 2006, 2007, 2008, 2009, 2010, 2011, 2012, 2013, 2014, 2015, 2016, 2018	DHS

Philippines	1993, 1998, 2003, 2008, 2013, 2017	DHS
Republic of Moldova	1997, 2005, 2012	RHS, MICS, DHS
Rwanda	2000, 2005, 2010, 2014	DHS
Sao Tome and Principe	2008, 2014	DHS, MICS
Senegal	1997, 2005, 2010, 2012, 2014, 2015, 2016, 2017	DHS
Serbia	2005, 2010, 2014	MICS
Sierra Leone	2005, 2008, 2010, 2013, 2017	DHS, MICS
South Africa	1998, 2016	DHS
Sudan	2010, 2014	MICS
Suriname	2006, 2010, 2018	MICS
Syrian Arab Republic	2006	MICS
Tajikistan	2005, 2012, 2017	DHS, MICS
Thailand	2005, 2012, 2015	MICS
Timor-Leste	2009, 2016	DHS
Togo	1998, 2006, 2010, 2013, 2017	DHS, MICS
Tunisia	2011, 2018	MICS
Turkey	1993, 1998, 2003, 2013	DHS
Uganda	1995, 2000, 2006, 2011, 2016	DHS
Ukraine	2005, 2007, 2012	DHS, MICS
Ukraine	2007, 2012	DHS, MICS
United Republic of Tanzania	1996, 1999, 2004, 2010, 2015	DHS
Uruguay	2012	MICS
Uzbekistan	1996, 2006	DHS, MICS
Vanuatu	2007	MICS
Viet Nam	1997, 2002, 2006, 2010, 2013	DHS, MICS
Yemen	2006, 2013	DHS, MICS
Zambia	1996, 2001, 2007, 2013, 2018	DHS
Zimbabwe	1994, 1999, 2005, 2009, 2010, 2014, 2015, 2019	DHS, MICS

Note:

1. Abbreviation: DHS - Demographic and Health Survey, MICS - Multiple Indicator Cluster Survey, RHS – Reproductive Health Survey

Table S2. Proportion of missing data in the indicators

**Indicator measures**	**% missing data**
**Beneficiary incidence in the poorest quintile [Q1] (%)**	
**All social protection and labour programmes**	46.3%
Social assistance	58.2%
Cash transfer	57.9%
Social insurance	59.3%
Labour market protection	72.5%
**Social protection coverage (%)**	
**All social protection and labour programmes**	47.2%
Social assistance	56.2%
Cash transfer	59.8%
Social insurance	59.3%
Labour market protection	72.5%
**Social protection coverage in Q1 (%)**	
**All social protection and labour programmes**	46.3%
Social assistance	58.2%
Cash transfer	56.8%
Social insurance	59.3%
Labour market protection	72.5%
**Social protection input per capita (US dollars)**	
**All social protection and labour programmes**	50.2%
Social assistance	54.8%
Cash transfer	60.1%
Social insurance	59.3%
Labour market protection	73.9%
**Social protection input per capita in Q1 (US dollars)**	
**All social protection and labour programmes**	46.2%
Social assistance	58.2%
Cash transfer	57.9%
Social insurance	59.3%
Labour market protection	72.5%
**Density of health professionals**	
Number of physicians per 1,000 people	37.5%
Number of nurses per 1,000 people	29.7%

Figure S1. Graphical comparisons between the distributions of the observed and the imputed values

A) Coverage of social protection and labour programme

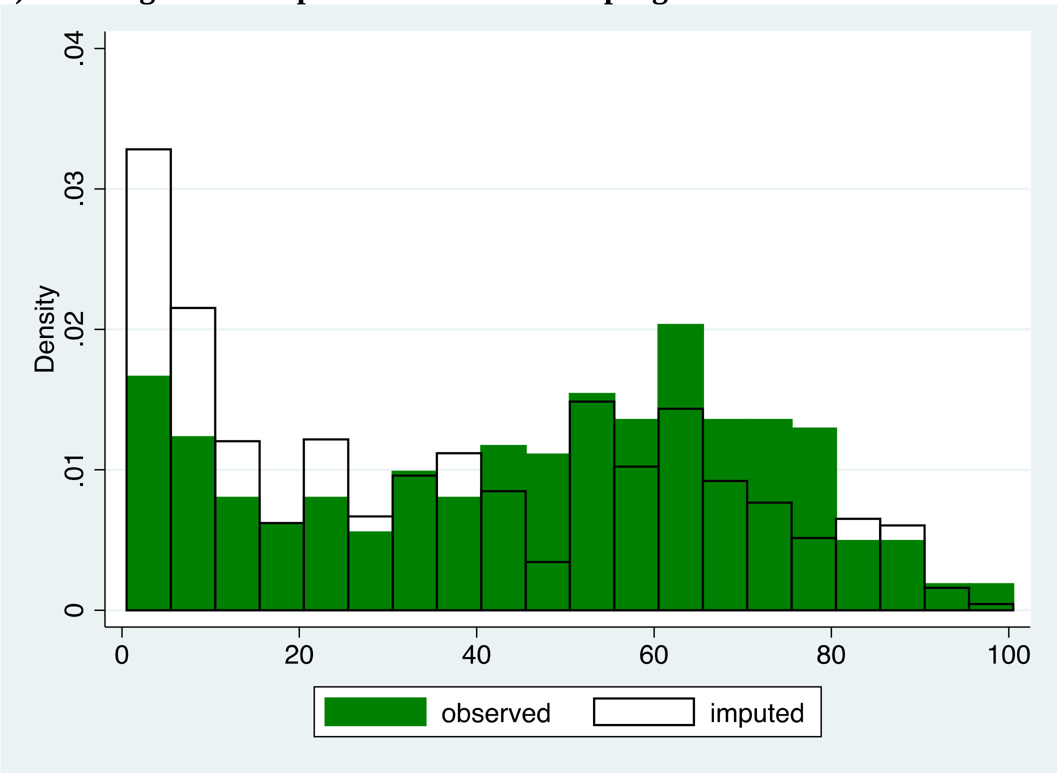

B) Coverage of social protection and labour programme in the poorest quintile

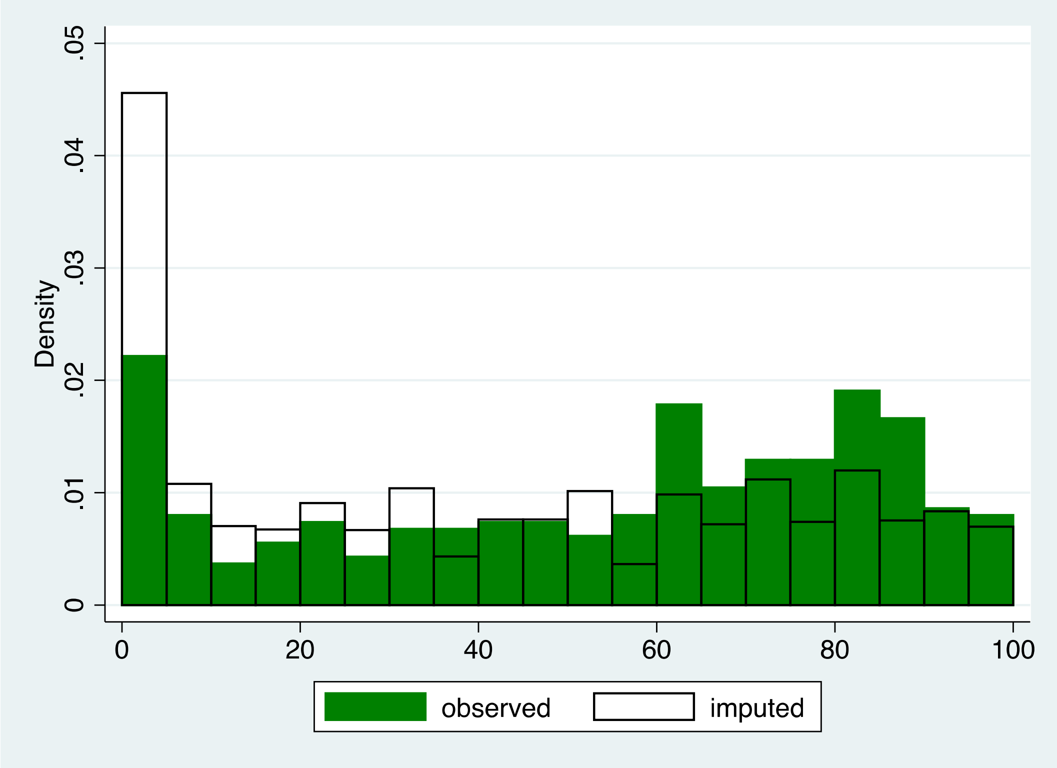

C) Beneficiary incidence social protection and labour programme in the poorest quintile

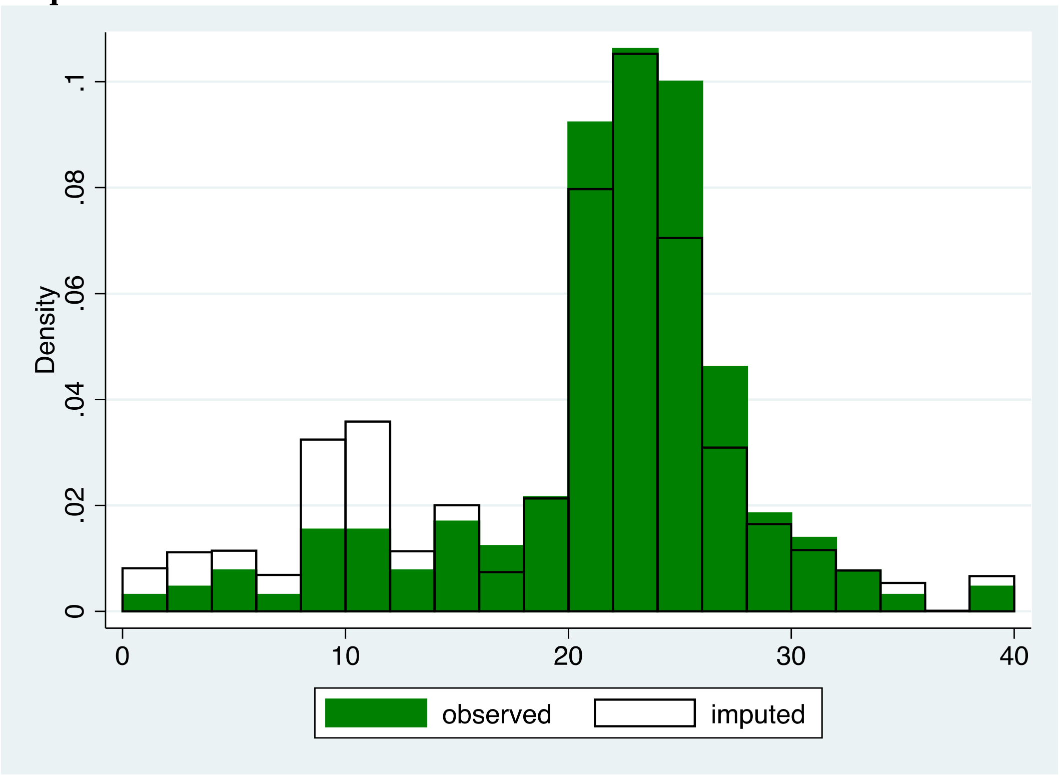

D) Input per beneficiary of social protection and labour programme

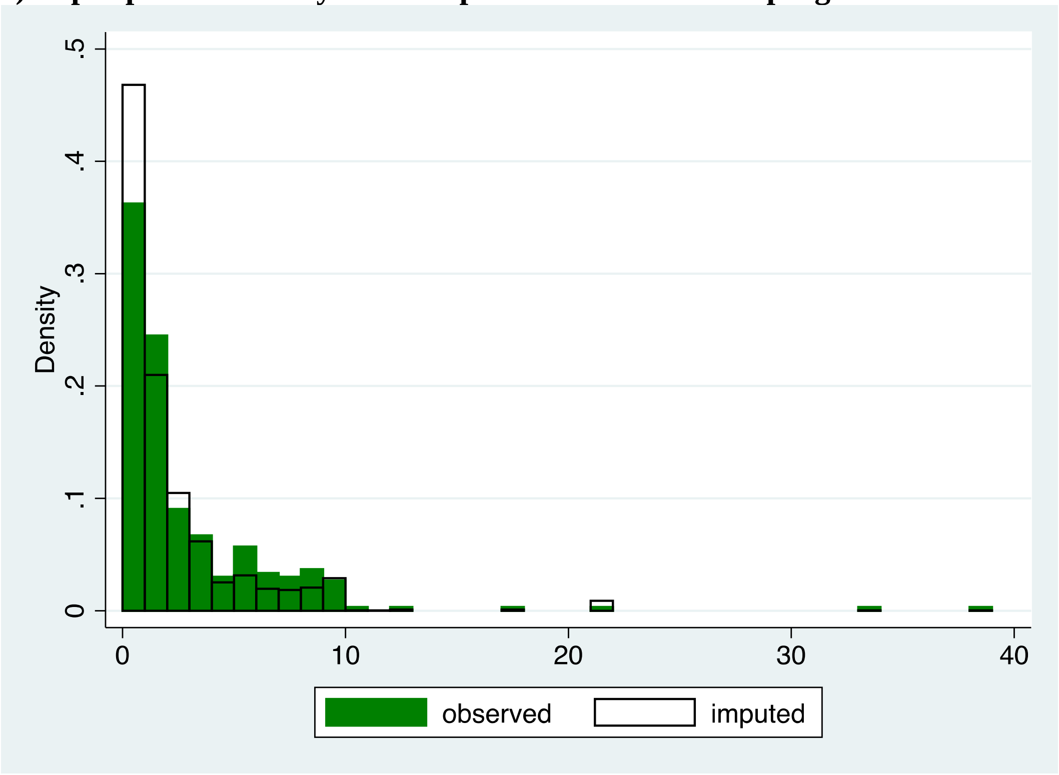

E) Input per beneficiary of social protection and labour programme in the poorest quintile

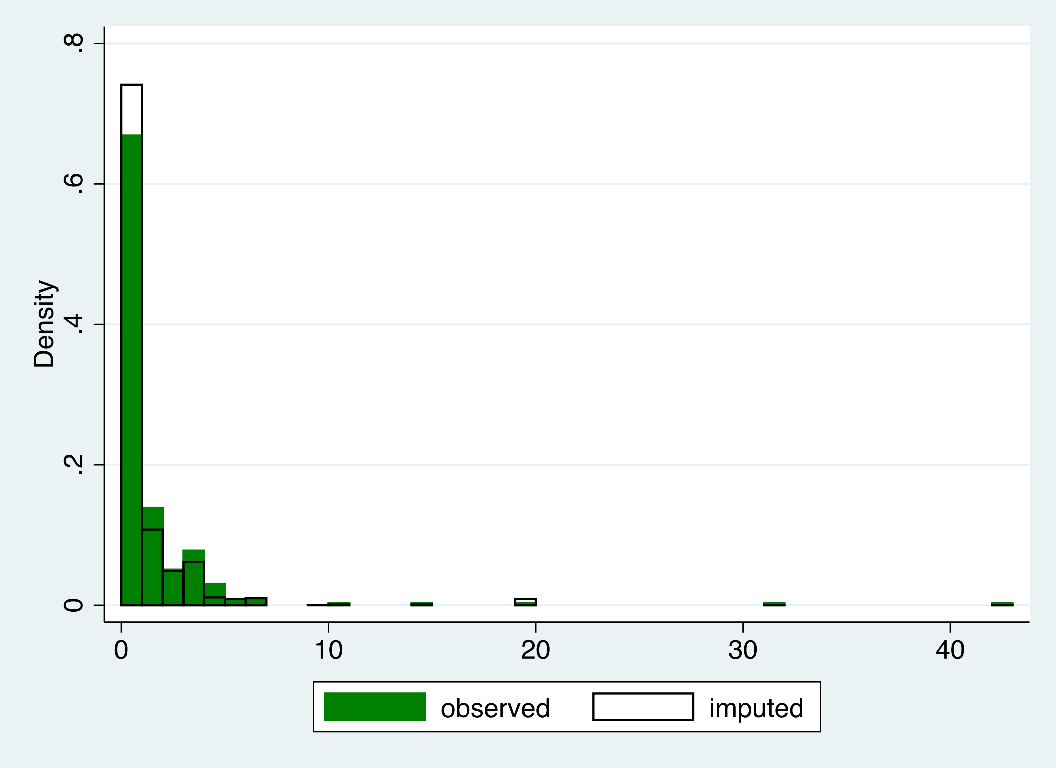

F) Number of physicians per 1,000 people

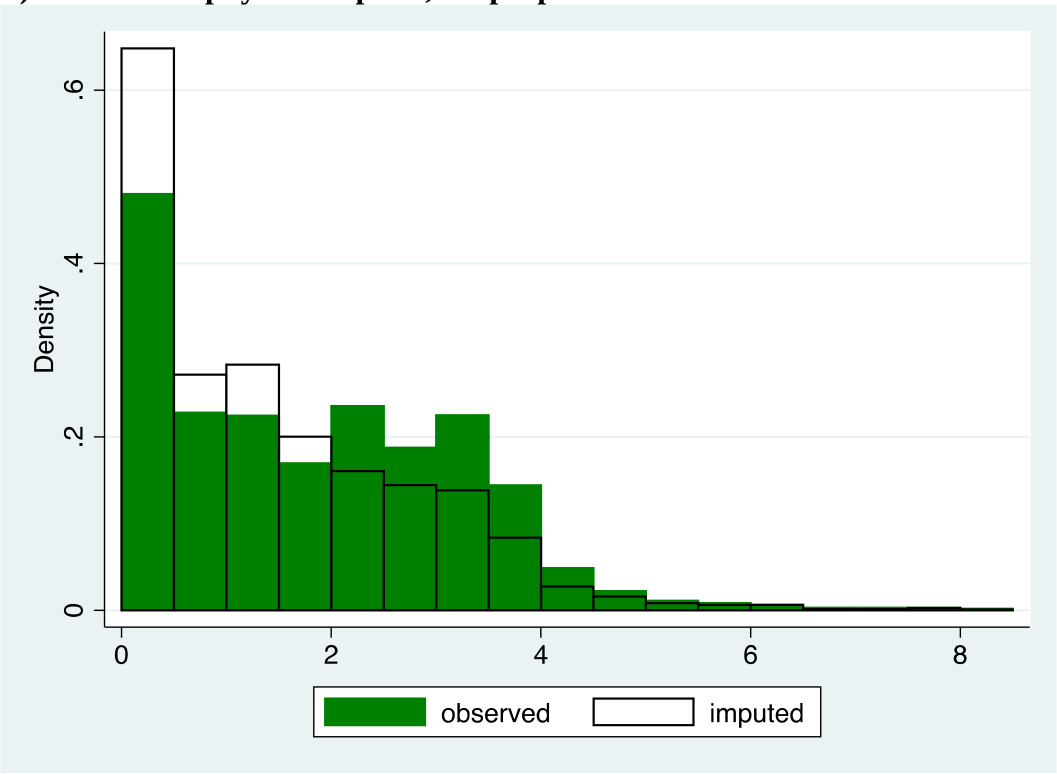

G) Number of nurses per 1,000 people

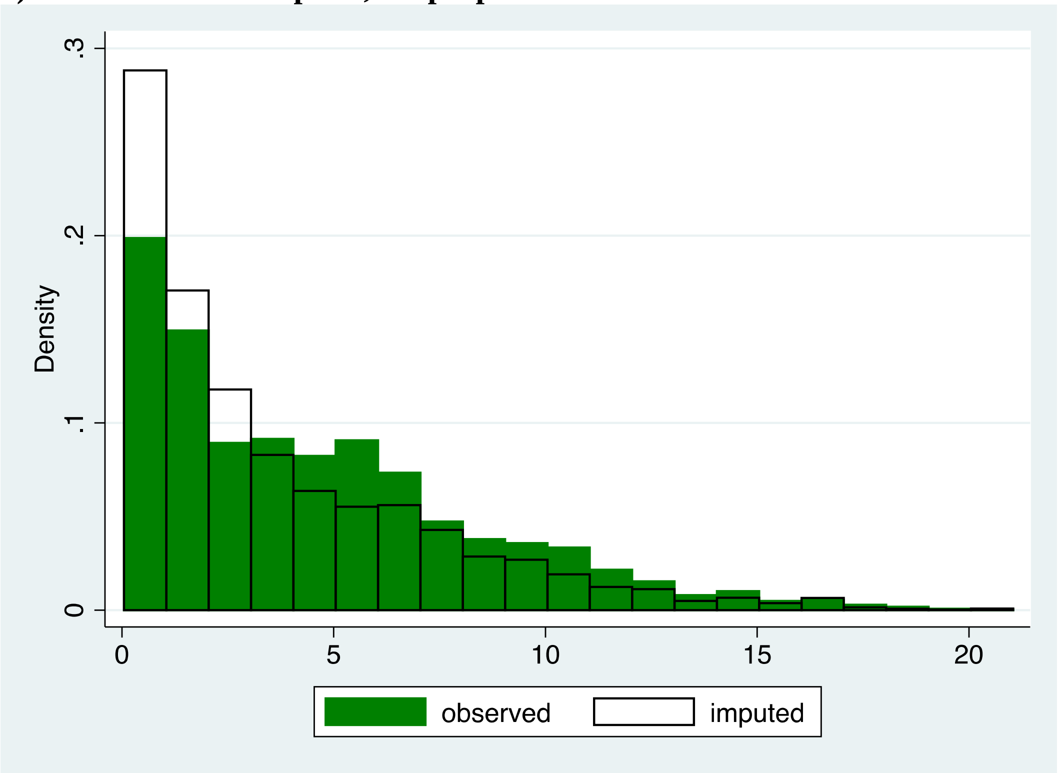

Figure S2. The relationship between the beneficiary incidence and input per beneficiary of social protection and labour (SPL) programme and under-5 mortality rate by quintile, using data from the most recent survey years

A) Beneficiary incidence of SPL in the poorest quintile (Q1) (%) and under-5 mortality rate

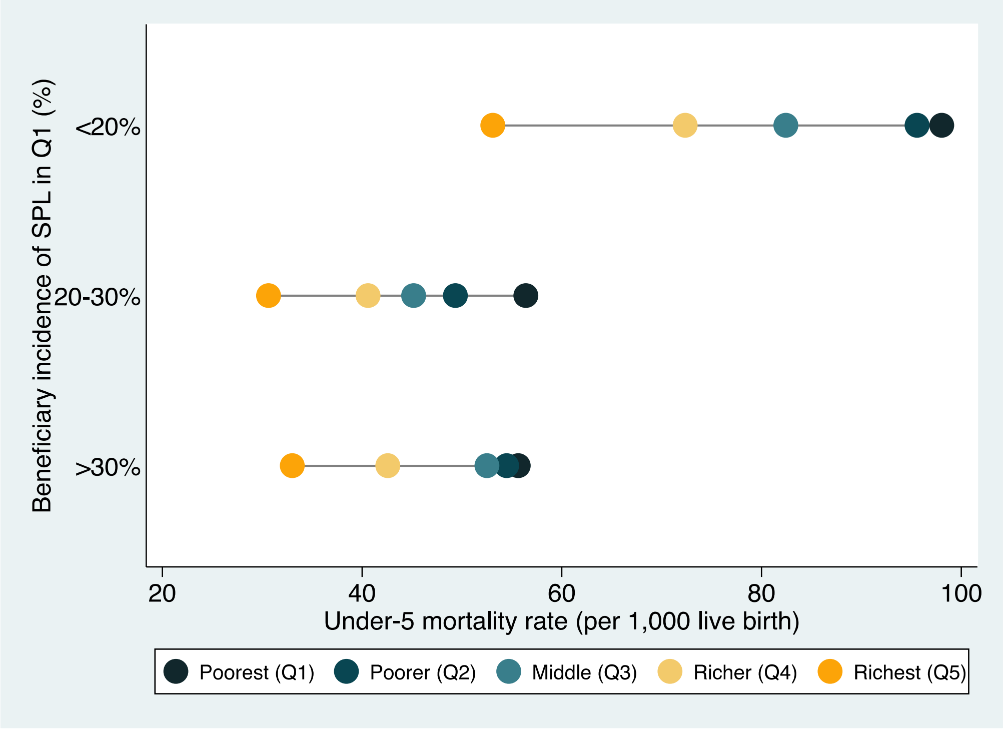

B) SPL input per beneficiary (US dollars) and under-5 mortality rate

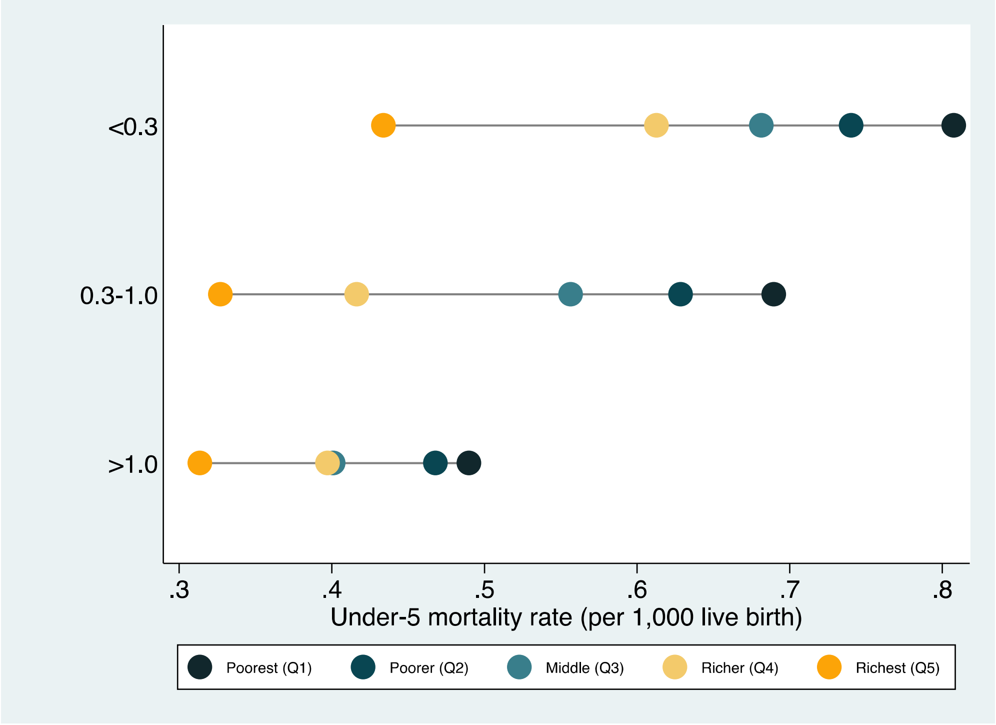

C) SPL input per beneficiary in Q1 (US dollars) and under-5 mortality rate

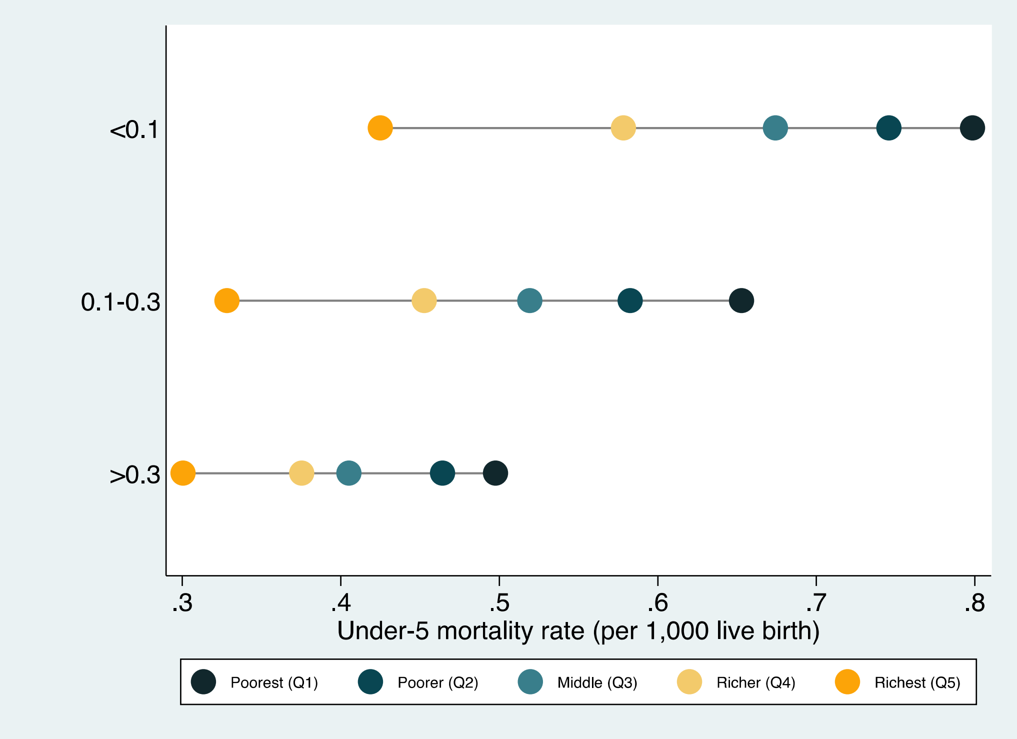

Figure S3. The relationship between the characteristics of social protection programmes and under-5 mortality rate by quintile, using data from the most recent survey years

A) Coverage of social protection programmes and under-5 mortality rate

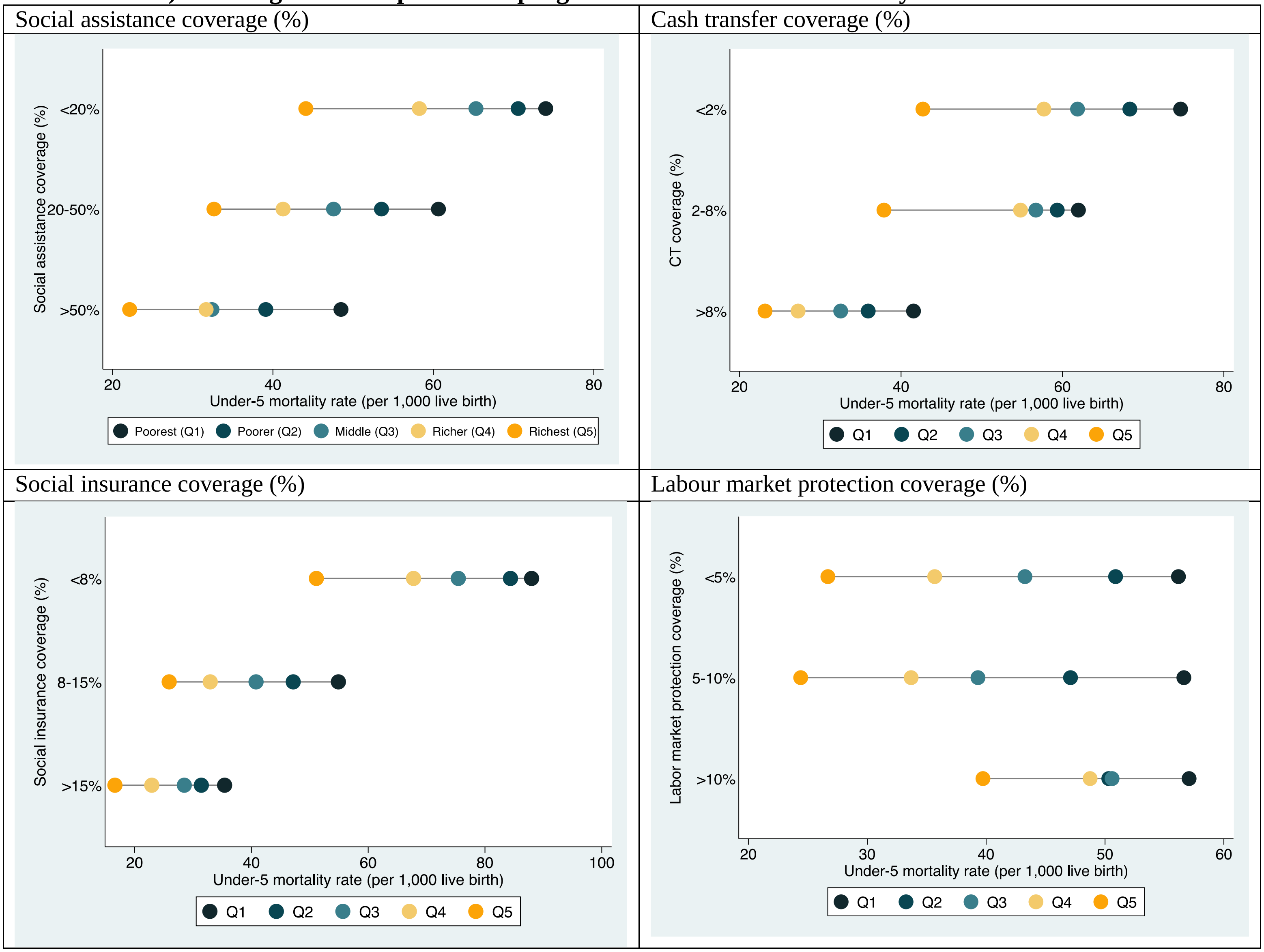

B) Coverage of social protection programmes in Q1 and under-5 mortality rate

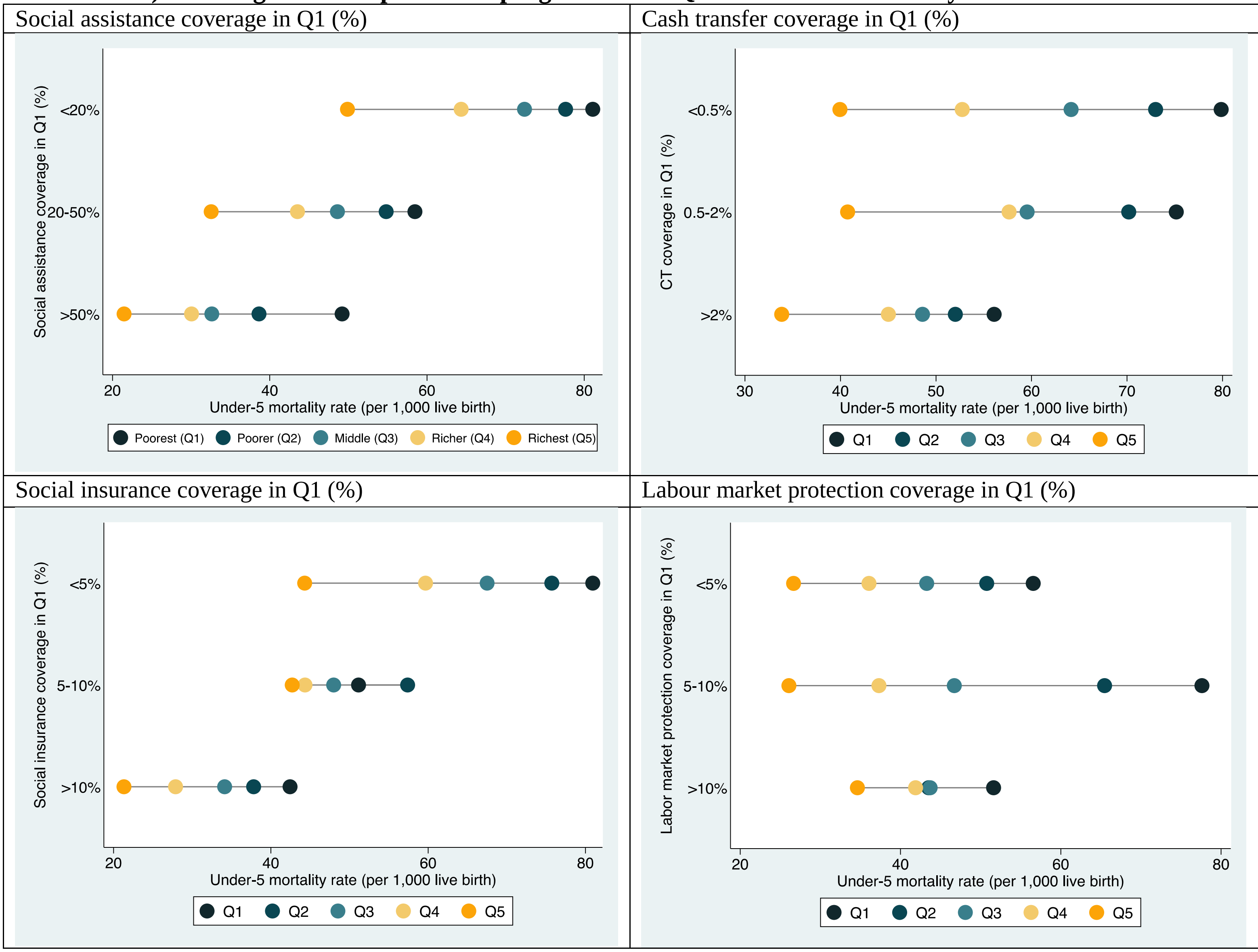

C) Beneficiary incidence in the poorest quintile (Q1) (%) and under-5 mortality rate

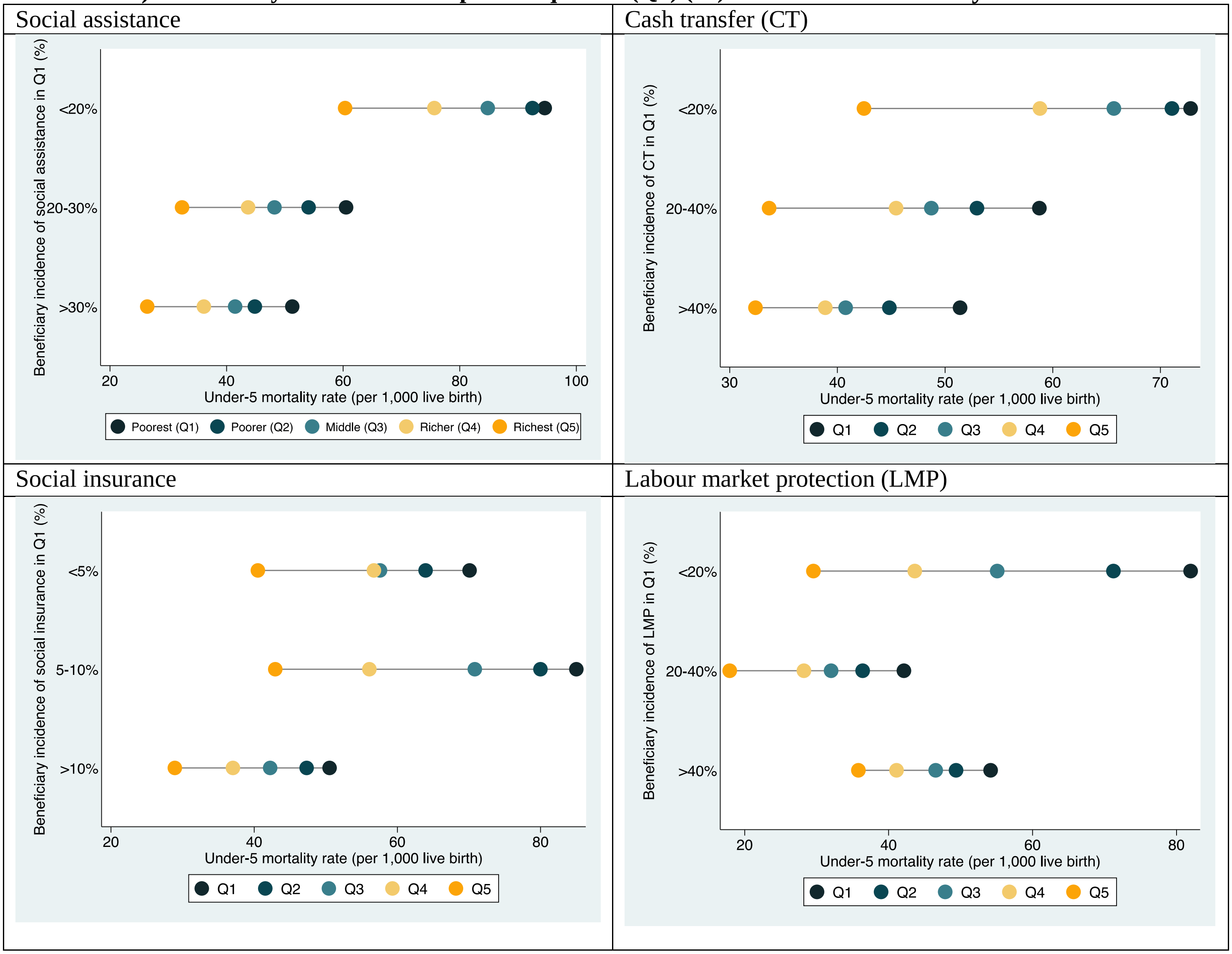

D) Social protection input per beneficiary (US dollars, $) and under-5 mortality rate

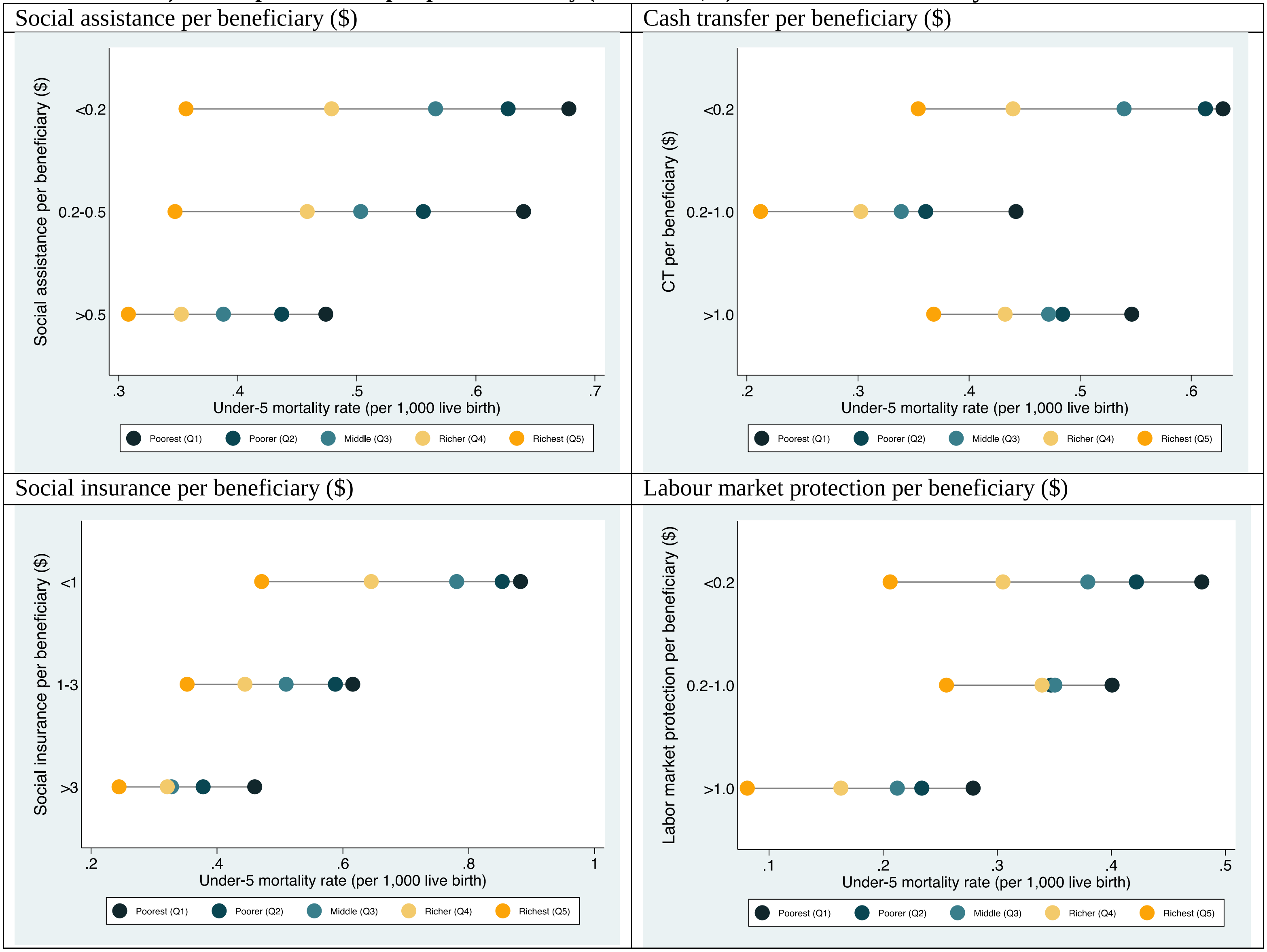

E) Social protection input per beneficiary in Q1 (US dollars, $) and under-5 mortality rate

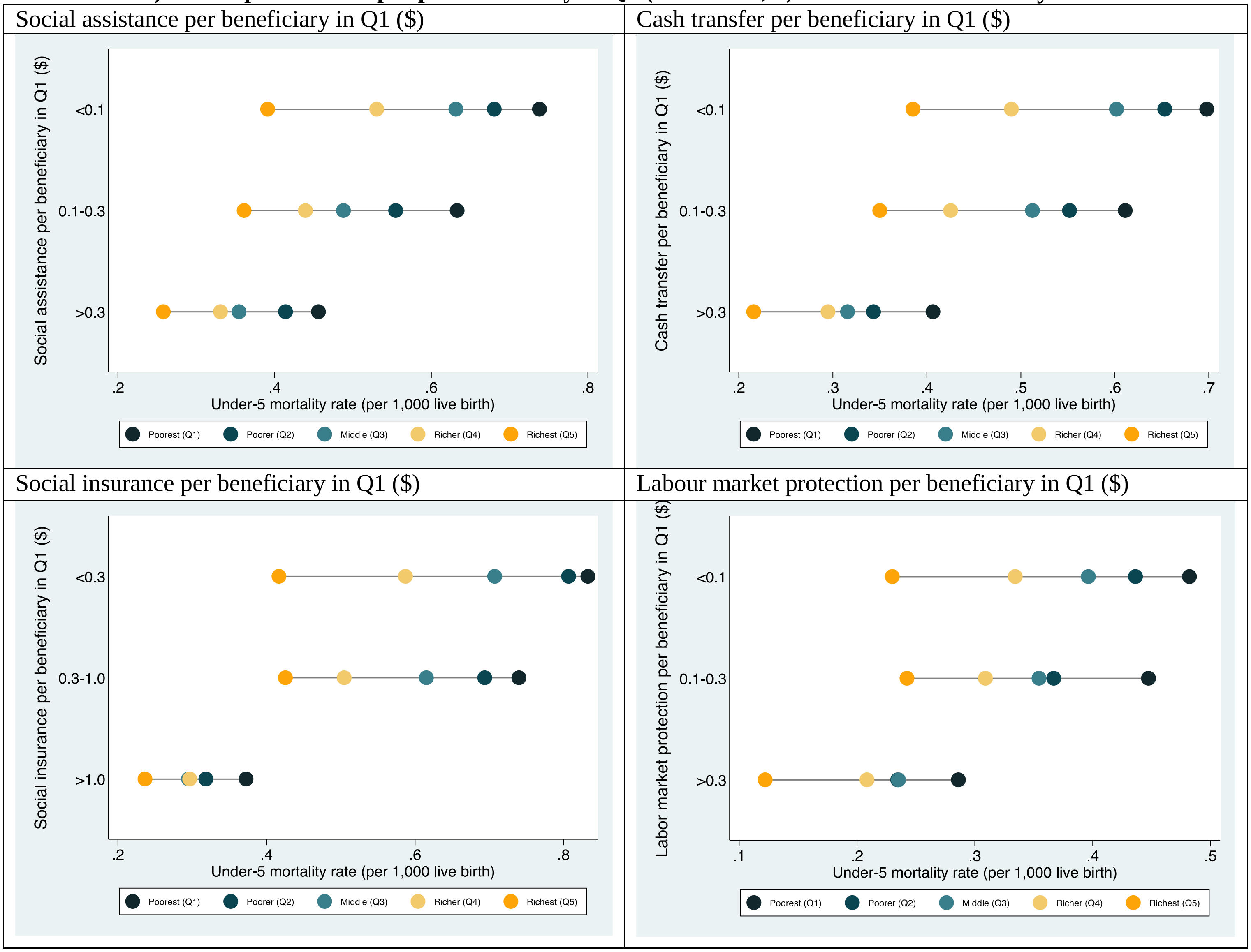

Table S3. Association between the beneficiary incidence and input per beneficiary of social protection and labour programmes and mortality indicators[1,2,3,4]

	All social protection and labour programmes	
	**Unadjusted**	**Adjusted**
**Beneficiary incidence in Q1 (%)**		
NMR	-0.42*** (-0.64, -0.20)	-0.09 (-0.25, 0.08)
PMR	-0.42*** (-0.64, -0.20)	-0.22* (-0.42, -0.03)
CMR	-1.14*** (-1.53, -0.75)	-0.75*** (-1.13, -0.37)
IMR	-0.84*** (-1.24, -0.44)	-0.30 (-0.63, 0.03)
U5MR	-1.90*** (-2.61, -1.19)	-1.00** (-1.60, -0.39)
**Social protection input per beneficiary ($)**		
NMR	-0.36** (-0.64, -0.09)	0.16 (-0.05, 0.36)
PMR	-0.21 (-0.51, 0.08)	0.25 (-0.03, 0.53)
CMR	-0.31 (-0.90, 0.27)	0.37 (-0.19, 0.93)
IMR	-0.58* (-1.10, -0.05)	0.41 (-0.03, 0.84)
U5MR	-0.88 (-1.89, 0.13)	0.82 (-0.05, 1.69)
**Social protection input per beneficiary in Q1 ($)**		
NMR	-0.41** (-0.68, -0.13)	0.19 (-0.03, 0.41)
PMR	-0.22 (-0.51, 0.08)	0.13 (-0.16, 0.42)
CMR	-0.42 (-1.01, 0.17)	0.51 (-0.08, 1.10)
IMR	-0.62* (-1.15, -0.09)	0.42 (-0.13, 0.97)
U5MR	-1.02* (-2.04, 0.00)	0.88 (-0.33, 2.08)

Note:

1. NMR: neonatal mortality rate, PMR: post-neonatal mortality rate, CMR: childhood mortality rate, IMR: infant mortality rate, U5MR: under-5 mortality rate
2. *** indicates *P* value<0.001; ** indicates *P* value<0.01; * indicates *P* value<0.05
3. In the adjusted model, we controlled for GDP per capita, current health expenditure as a percentage of GDP, public health expenditure as a percentage of GDP, physician density per 1,000 people, nurse density per 1,000 people, and population size. In the sensitivity analysis, besides all the covariates controlled in the adjusted model, we further controlled for country-fixed effects
4. When analyzing the association between social protection input per beneficiary and health indicators, we divided the values of the health indicators by 10. So the coefficient β could be interpreted as when the social protection input per beneficiary increased by 10%, the mortality rate would change by β per 1,000 live births.

Table S4. Association between the subtypes of social protection programmes and mortality indicators[1,2]

	Neonatal mortality rate		Post-neonatal mortality rate	
	Unadjusted	Adjusted	Unadjusted	Adjusted
**Social protection coverage (%)**				
Social assistance	-0.15*** (-0.22, -0.07)	-0.08*** (-0.11, -0.05)	-0.15*** (-0.22, -0.07)	-0.10*** (-0.13, -0.06)
Cash transfer	-0.16* (-0.30, -0.02)	-0.07* (-0.13, -0.00)	-0.17* (-0.31, -0.04)	-0.02 (-0.09, 0.04)
Social insurance	-0.30*** (-0.40, -0.20)	-0.14*** (-0.21, -0.07)	-0.28*** (-0.38, -0.17)	-0.10* (-0.19, -0.02)
Labour market protection	-0.16 (-0.62, 0.30)	0.04 (-0.14, 0.22)	-0.17 (-0.57, 0.24)	0.01 (-0.19, 0.20)
**Social protection coverage in Q1 (%)**				
Social assistance	-0.15*** (-0.21, -0.09)	-0.08*** (-0.11, -0.05)	-0.14*** (-0.20, -0.08)	-0.09*** (-0.12, -0.05)
Cash transfer	-0.22*** (-0.33, -0.11)	-0.04 (-0.09, 0.01)	-0.21*** (-0.33, -0.10)	-0.01 (-0.07, 0.06)
Social insurance	-0.24*** (-0.34, -0.14)	-0.09** (-0.16, -0.03)	-0.22*** (-0.32, -0.12)	-0.06 (-0.14, 0.01)
Labour market protection	-0.28 (-0.70, 0.15)	-0.07 (-0.25, 0.10)	-0.24 (-0.61, 0.14)	-0.08 (-0.27, 0.11)
**Beneficiary incidence in Q1 (%)**				
Social assistance	-0.39*** (-0.59, -0.18)	-0.17*** (-0.24, -0.10)	-0.48*** (-0.68, -0.27)	-0.17* (-0.30, -0.04)
Cash transfer	-0.10 (-0.29, 0.08)	-0.06 (-0.53, 0.41)	-0.11 (-0.29, 0.06)	0.15** (0.06, 0.23)
Social insurance	-0.32** (-0.53, -0.11)	-0.15* (-0.28, -0.01)	-0.24* (-0.45, -0.04)	-0.07 (-0.17, 0.04)
Labour market protection	-0.17 (-0.39, 0.05)	-0.07 (-0.24, 0.10)	-0.27*** (-0.34, -0.20)	-0.20* (-0.40, -0.01)
**Social protection input per beneficiary (US dollars)**				
Social assistance	0.06 (-0.37, 0.49)	0.02 (-0.09, 0.13)	0.27 (-0.19, 0.72)	0.39 (-0.02, 0.79)
Cash transfer	-0.16 (-0.76, 0.43)	0.03 (-0.24, 0.29)	0.28 (-0.23, 0.79)	0.40 (-0.11, 0.91)
Social insurance	-0.43*** (-0.67, -0.19)	-0.01 (-0.12, 0.11)	-0.24* (-0.48, -0.01)	0.12 (-0.01, 0.25)
Labour market protection	-0.36** (-0.64, -0.09)	1.60 (1.60, 1.60)	-0.21 (-0.51, 0.08)	0.02 (-0.25, 0.28)
**Social protection input per beneficiary in Q1 (US dollars)**				
Social assistance	0.15** (0.06, 0.23)	0.10 (-0.13, 0.33)	0.18 (-0.32, 0.68)	0.38 (-0.15, 0.91)
Cash transfer	-6.56* (-12.17, -0.94)	8.14 (8.14, 8.14)	-1.44 (-4.12, 1.24)	0.37 (-0.14, 0.88)
Social insurance	-0.42** (-0.67, -0.17)	0.10 (-0.02, 0.23)	-0.21 (-0.46, 0.04)	0.29 (-0.05, 0.63)
Labour market protection	-0.41** (-0.68, -0.13)	0.10 (-0.10, 0.31)	-0.22 (-0.51, 0.08)	0.51(-0.10, 1.12)

Note:

1. *** indicates *P* value<0.001; ** indicates *P* value<0.01; * indicates *P* value<0.05
2. When analyzing the association between social protection input per beneficiary and health indicators, we divided the values of the health indicators by 10. So the coefficient β could be interpreted as when the social protection input per beneficiary increased by 10%, the mortality rate would change by β per 1,000 live births.

Table S4. Association between the subtypes of social protection programmes and mortality indicators (continued)

	Childhood mortality rate		Infant mortality rate	
	**Unadjusted**	**Adjusted**	**Unadjusted**	**Adjusted**
**Social protection coverage (%)**				
Social assistance	-0.30*** (-0.44, -0.17)	-0.22*** (-0.29, -0.14)	-0.29*** (-0.43, -0.16)	-0.18*** (-0.24, -0.12)
Cash transfer	-0.23 (-0.46, 0.00)	0.02 (-0.11, 0.15)	-0.33** (-0.59, -0.08)	-0.09 (-0.20, 0.03)
Social insurance	-0.54*** (-0.76, -0.31)	-0.18 (-0.38, 0.01)	-0.58*** (-0.77, -0.39)	-0.25*** (-0.39, -0.11)
Labour market protection	-0.07 (-0.63, 0.49)	0.12 (-0.15, 0.39)	-0.33 (-1.15, 0.50)	0.05 (-0.29, 0.39)
**Social protection coverage in Q1 (%)**				
Social assistance	-0.29*** (-0.40, -0.18)	-0.22*** (-0.28, -0.15)	-0.29*** (-0.40, -0.18)	-0.17*** (-0.22, -0.12)
Cash transfer	-0.35** (-0.57, -0.14)	-0.02 (-0.15, 0.11)	-0.43*** (-0.64, -0.23)	-0.05 (-0.15, 0.06)
Social insurance	-0.43*** (-0.65, -0.22)	-0.12 (-0.29, 0.06)	-0.45*** (-0.64, -0.27)	-0.16* (-0.28, -0.03)
Labour market protection	-0.12 (-0.65, 0.40)	0.12 (-0.14, 0.39)	-0.51 (-1.27, 0.25)	-0.15 (-0.49, 0.18)
**Beneficiary incidence in Q1 (%)**				
Social assistance	-1.03*** (-1.39, -0.67)	-0.89*** (-1.13, -0.64)	-0.86*** (-1.24, -0.49)	-0.15 (-0.37, 0.07)
Cash transfer	-0.20 (-0.51, 0.10)	0.07 (-0.11, 0.24)	-0.22 (-0.55, 0.12)	0.29 (-0.06, 0.65)
Social insurance	-0.52* (-0.96, -0.08)	-0.16 (-0.40, 0.08)	-0.56** (-0.95, -0.17)	-0.21 (-0.49, 0.06)
Labour market protection	-0.23*** (-0.33, -0.12)	-0.16 (-0.43, 0.12)	-0.37 (-0.77, 0.03)	-0.43 (-0.96, 0.09)
**Social protection input per beneficiary (US dollars)**				
Social assistance	0.26 (-0.61, 1.13)	0.43 (-0.10, 0.96)	0.68* (0.07, 1.29)	0.33 (-0.49, 1.15)
Cash transfer	-1.93** (-3.17, -0.69)	0.78 (-0.46, 2.03)	-8.55** (-14.16, -2.94)	0.43 (-0.01, 0.87)
Social insurance	-0.56* (-1.09, -0.03)	0.13 (-0.19, 0.45)	-0.67** (-1.13, -0.20)	0.11 (-0.11, 0.33)
Labour market protection	-0.31 (-0.90, 0.27)	12.42 (12.42, 12.42)	-0.58* (-1.10, -0.05)	9.74 (9.74, 9.74)
**Social protection input per beneficiary in Q1 (US dollars)**				
Social assistance	0.68** (0.20, 1.15)	0.21 (-0.73, 1.16)	0.68*** (0.33, 1.02)	0.12 (-0.77, 1.01)
Cash transfer	-1.90* (-3.66, -0.14)	0.81*** (0.57, 1.05)	-8.00 (-16.09, 0.10)	0.39 (-0.05, 0.83)
Social insurance	-0.52 (-1.07, 0.03)	0.44 (-0.01, 0.89)	-0.63** (-1.10, -0.16)	0.40 (-0.04, 0.84)
Labour market protection	-0.42 (-1.01, 0.17)	0.78 (-0.17, 1.73)	-0.62* (-1.15, -0.09)	0.51 (-0.10, 1.12)

Table S4. Association between the subtypes of social protection programmes and mortality indicators (continued)

	Under-5 mortality rate	
	**Unadjusted**	**Adjusted**
**Social protection coverage (%)**		
Social assistance	-0.57*** (-0.82, -0.33)	-0.38*** (-0.50, -0.27)
Cash transfer	-0.55* (-0.97, -0.12)	-0.07 (-0.27, 0.13)
Social insurance	-1.08*** (-1.46, -0.71)	-0.41** (-0.71, -0.12)
Labour market protection	-0.40 (-1.68, 0.87)	0.15 (-0.38, 0.68)
**Social protection coverage in Q1 (%)**		
Social assistance	-0.56*** (-0.75, -0.37)	-0.37*** (-0.47, -0.27)
Cash transfer	-0.76*** (-1.14, -0.39)	-0.07 (-0.27, 0.14)
Social insurance	-0.86*** (-1.22, -0.50)	-0.26 (-0.52, 0.00)
Labour market protection	-0.64 (-1.82, 0.55)	-0.05 (-0.57, 0.47)
**Beneficiary incidence in Q1 (%)**		
Social assistance	-1.82*** (-2.47, -1.17)	-0.97*** (-1.37, -0.57)
Cash transfer	-0.41 (-0.97, 0.15)	0.35 (-0.02, 0.72)
Social insurance	-1.05** (-1.81, -0.29)	-0.35 (-0.72, 0.01)
Labour market protection	-0.52 (-1.14, 0.10)	-0.64*** (-0.84, -0.45)
**Social protection input per beneficiary (US dollars)**		
Social assistance	0.56 (-0.98, 2.09)	1.16 (-0.49, 2.80)
Cash transfer	-10.35** (-17.02, -3.67)	1.13 (-0.08, 2.35)
Social insurance	-1.19* (-2.10, -0.29)	0.22 (-0.26, 0.71)
Labour market protection	-0.88 (-1.89, 0.13)	21.61 (21.61, 21.61)
**Social protection input per beneficiary in Q1 (US dollars)**		
Social assistance	0.30 (-1.37, 1.97)	1.29 (-0.42, 3.01)
Cash transfer	-9.78* (-19.42, -0.15)	1.12 (-0.09, 2.34)
Social insurance	-1.12* (-2.06, -0.19)	0.79 (-0.73, 2.32)
Labour market protection	-1.02 (-2.04, 0.00)	1.35 (-0.13, 2.83)

Table S5. Association between the beneficiary incidence and input per beneficiary of social protection programmes and concentration index of mortality indicators[1]

	All social protection and labour programmes			
	Unadjusted		Adjusted	
	**Coefficient**	**Reducing inequalities**	**Coefficient**	**Reducing inequalities**
**Beneficiary incidence in Q1 (%)**				
NMR	0.30 (-0.00, 0.60)	No	-0.17 (-0.37, 0.02)	No
PMR	0.29 (0.09, 0.49)	Yes	0.24 (0.01, 0.47)	Yes
CMR	0.47 (0.17, 0.76)	Yes	0.27 (0.04, 0.51)	Yes
IMR	-0.07 (-0.28, 0.13)	No	0.03 (-0.16, 0.21)	No
U5MR	0.08 (0.03, 0.13)	Yes	0.08 (-0.10, 0.25)	No
**Social protection input per beneficiary (US dollars)**				
NMR	-0.10 (-0.37, 0.16)	No	0.16 (-0.12, 0.43)	No
PMR	-0.18 (-0.55, 0.18)	No	-0.17 (-0.37, 0.02)	No
CMR	-0.21 (-0.56, 0.15)	No	0.27 (-0.06, 0.59)	No
IMR	-0.12 (-0.38, 0.14)	No	0.18 (-0.06, 0.43)	No
U5MR	-0.09 (-0.34, 0.17)	No	0.21 (-0.03, 0.44)	No
**Social protection input per beneficiary in Q1 (US dollars)**				
NMR	-0.15 (-0.42, 0.12)	No	0.12 (-0.18, 0.41)	No
PMR	-0.07 (-0.44, 0.30)	No	0.17 (-0.02, 0.37)	No
CMR	-0.23 (-0.59, 0.12)	No	0.28 (-0.05, 0.62)	No
IMR	-0.10 (-0.36, 0.17)	No	0.25 (-0.01, 0.51)	No
U5MR	-0.07 (-0.33, 0.19)	No	0.25 (-0.02, 0.51)	No

Note:

1. NMR: neonatal mortality rate, PMR: post-neonatal mortality rate, CMR: childhood mortality rate, IMR: infant mortality rate, U5MR: under-5 mortality rate, No: no significant change

Table S6. Association between social protection programmes and slope index of inequality[1]

	All social protection and labour programmes			
	Unadjusted		Adjusted	
	Coefficient	Reducing inequalities	Coefficient	Reducing inequalities
**Social protection coverage (%)**				
NMR	-0.03 (-0.10, 0.03)	No	-0.06 (-0.15, 0.03)	No
PMR	0.09 (0.02, 0.16)	Yes	0.11 (0.02, 0.20)	Yes
CMR	0.27 (0.15, 0.38)	Yes	0.20 (0.06, 0.34)	Yes
IMR	0.05 (-0.07, 0.16)	No	0.04 (-0.11, 0.19)	No
U5MR	0.27 (0.09, 0.46)	Yes	0.21 (-0.03, 0.44)	No
**Social protection coverage in Q1 (%)**				
NMR	-0.04 (-0.09, 0.02)	No	-0.06 (-0.14, 0.01)	No
PMR	0.08 (0.02, 0.14)	Yes	0.09 (0.02, 0.16)	Yes
CMR	0.25 (0.15, 0.35)	Yes	0.20 (0.08, 0.32)	Yes
IMR	0.03 (-0.07, 0.13)	No	0.01 (-0.12, 0.14)	No
U5MR	0.24 (0.08, 0.40)	Yes	0.17 (-0.03, 0.38)	No
**Beneficiary incidence in Q1 (%)**				
NMR	-0.07 (-0.31, 0.17)	No	-0.15 (-0.41, 0.10)	No
PMR	0.56 (0.31, 0.81)	Yes	0.52 (0.25, 0.78)	Yes
CMR	1.16 (0.75, 1.57)	Yes	0.83 (0.41, 1.25)	Yes
IMR	0.35 (-0.05, 0.74)	No	0.20 (-0.23, 0.64)	No
U5MR	1.18 (0.54, 1.82)	Yes	0.73 (0.06, 1.41)	Yes
**Social protection input per beneficiary (US dollars)**				
NMR	0.23 (-0.06, 0.51)	No	0.23 (-0.10, 0.56)	No
PMR	0.22 (-0.07, 0.51)	No	0.12 (-0.21, 0.45)	No
CMR	0.57 (0.01, 1.12)	Yes	-0.00 (-0.62, 0.61)	No
IMR	0.46 (0.02, 0.91)	Yes	0.41 (-0.11, 0.93)	No
U5MR	1.02 (0.20, 1.83)	Yes	0.52 (-0.40, 1.44)	No
**Social protection input per beneficiary in Q1 (US dollars)**				

NMR	0.16 (-0.13, 0.45)	No	0.13 (-0.22, 0.49)	No
PMR	0.27 (-0.01, 0.56)	No	0.17 (-0.17, 0.52)	No
CMR	0.60 (0.04, 1.16)	Yes	-0.03 (-0.66, 0.60)	No
IMR	0.43 (-0.01, 0.88)	No	0.31 (-0.23, 0.85)	No
U5MR	0.99 (0.18, 1.81)	Yes	0.30 (-0.64, 1.24)	No

Note:

1. NMR: neonatal mortality rate, PMR: post-neonatal mortality rate, CMR: childhood mortality rate, IMR: infant mortality rate, U5MR: under-5 mortality rate, No: no significant change

Table S7. Association between social protection programmes and difference of mortality indicators between the richest (Q5) and the poorest (Q1) quintiles[1]

	All social protection and labour programmes			
	Unadjusted		Adjusted	
	Coefficient	Reducing inequalities	Coefficient	Reducing inequalities
**Social protection coverage (%)**				
NMR	-0.03 (-0.10, 0.05)	No	-0.06 (-0.16, 0.04)	No
PMR	0.08 (-0.00, 0.16)	No	0.09 (-0.02, 0.20)	No
CMR	0.23 (0.10, 0.36)	Yes	0.18 (0.02, 0.34)	Yes
IMR	0.05 (-0.08, 0.19)	No	0.03 (-0.15, 0.21)	No
U5MR	0.26 (0.05, 0.48)	Yes	0.19 (-0.09, 0.46)	No
**Social protection coverage in Q1 (%)**				
NMR	-0.03 (-0.09, 0.04)	No	-0.06 (-0.15, 0.03)	No
PMR	0.07 (-0.01, 0.14)	No	0.07 (-0.03, 0.16)	No
CMR	0.22 (0.10, 0.33)	Yes	0.17 (0.03, 0.31)	Yes
IMR	0.04 (-0.08, 0.15)	No	0.01 (-0.15, 0.16)	No
U5MR	0.23 (0.05, 0.42)	Yes	0.16 (-0.07, 0.39)	No
**Beneficiary incidence in Q1 (%)**				
NMR	-0.04 (-0.32, 0.25)	No	-0.11 (-0.42, 0.20)	No
PMR	0.37 (0.08, 0.67)	Yes	0.33 (0.00, 0.67)	Yes
CMR	0.95 (0.48, 1.42)	Yes	0.68 (0.19, 1.17)	Yes
IMR	0.34 (-0.14, 0.82)	No	0.22 (-0.32, 0.77)	No
U5MR	1.21 (0.44, 1.97)	Yes	0.85 (0.03, 1.66)	Yes
**Social protection input per beneficiary (US dollars)**				
NMR	0.10 (-0.22, 0.43)	No	0.07 (-0.34, 0.47)	No
PMR	0.23 (-0.12, 0.57)	No	0.18 (-0.25, 0.60)	No
CMR	0.51 (-0.13, 1.15)	No	0.03 (-0.70, 0.75)	No
IMR	0.33 (-0.20, 0.86)	No	0.24 (-0.42, 0.91)	No
U5MR	0.81 (-0.14, 1.76)	No	0.29 (-0.82, 1.39)	No

**Social protection input per beneficiary in Q1 (US dollars)**				
NMR	0.08 (-0.25, 0.41)	No	0.04 (-0.38, 0.45)	No
PMR	0.23 (-0.12, 0.57)	No	0.17 (-0.27, 0.61)	No
CMR	0.53 (-0.11, 1.18)	No	-0.01 (-0.75, 0.74)	No
IMR	0.31 (-0.22, 0.85)	No	0.20 (-0.48, 0.89)	No
U5MR	0.81 (-0.15, 1.76)	No	0.22 (-0.92, 1.35)	No

Note:

1. NMR: neonatal mortality rate, PMR: post-neonatal mortality rate, CMR: childhood mortality rate, IMR: infant mortality rate, U5MR: under-5 mortality rate, No: no significant change

Table S8. Sensitivity analysis on the association between the coverage of social protection and labour programmes and health outcome indicators[1,2,3]

	All social protection and labour programmes
**Social protection coverage (%)**	
NMR	-0.09*** (-0.13, -0.04)
PMR	-0.11** (-0.18, -0.05)
CMR	-0.20*** (-0.33, -0.07)
IMR	-0.21*** (-0.32, -0.10)
U5MR	-0.44*** (-0.60, -0.28)
**Social protection coverage in Q1 (%)**	
NMR	-0.09*** (-0.15, -0.03)
PMR	-0.09** (-0.15, -0.04)
CMR	-0.28*** (-0.39, -0.16)
IMR	-0.18*** (-0.27, -0.09)
U5MR	-0.35*** (-0.49, -0.20)
**Beneficiary incidence in Q1 (%)**	
NMR	-0.08 (-0.25, 0.10)
PMR	-0.19* (-0.38, -0.01)
CMR	-0.78*** (-1.17, -0.39)
IMR	-0.27 (-0.60, 0.06)
U5MR	-0.99** (-1.61, -0.38)
**Social protection input per beneficiary (US dollars)**	
NMR	0.18 (-0.03, 0.39)
PMR	-0.21 (-0.50, 0.09)
CMR	-0.33 (-0.92, 0.25)
IMR	-0.48 (-1.11, 0.15)
U5MR	-0.90 (-1.92, 0.12)
**Social protection input per beneficiary in Q1 (US dollars)**	
NMR	0.22 (-0.00, 0.45)
PMR	-0.21 (-0.51, 0.09)
CMR	-0.44 (-1.03, 0.16)
IMR	-0.52 (-1.16, 0.11)
U5MR	-0.94 (-2.08, 0.19)

Note:

1. NMR: neonatal mortality rate, PMR: post-neonatal mortality rate, CMR: childhood mortality rate, IMR: infant mortality rate, U5MR: under-5 mortality rate
2. *** indicates *P* value<0.001; ** indicates *P* value<0.01; * indicates *P* value<0.05
3. When analyzing the association between social protection input per beneficiary and health indicators, we divided the values of the health indicators by 10. So the coefficient β could be interpreted as when the social protection input per beneficiary increased by 10%, the mortality rate would change by β per 1,000 live births.

Table S9. Sensitivity analysis on the association between the subtypes of social protection programmes and mortality indicators[1,2,3]

	NMR	PMR	CMR	IMR	U5MR
**Social protection coverage (%)**					
Social assistance	-0.08** (-0.14, -0.02)	-0.09** (-0.16, -0.02)	-0.22** (-0.35, -0.09)	-0.17** (-0.28, -0.06)	-0.37*** (-0.58, -0.17)
Cash transfer	-0.07 (-0.18, 0.05)	-0.02 (-0.14, 0.11)	0.02 (-0.22, 0.25)	-0.08 (-0.29, 0.13)	-0.07 (-0.43, 0.30)
Social insurance	-0.14* (-0.27, -0.02)	-0.10 (-0.25, 0.05)	-0.18 (-0.53, 0.17)	-0.24 (-0.49, 0.01)	-0.41 (-0.94, 0.12)
Labour market protection	0.07 (-0.27, 0.40)	0.08 (-0.31, 0.48)	0.22 (-0.32, 0.75)	0.15 (-0.51, 0.82)	0.34 (-0.70, 1.38)
**Social protection coverage in Q1 (%)**					
Social assistance	-0.08** (-0.13, -0.03)	-0.09** (-0.15, -0.03)	-0.22*** (-0.33, -0.10)	-0.17*** (-0.27, -0.07)	-0.37*** (-0.55, -0.20)
Cash transfer	-0.04 (-0.14, 0.06)	-0.01 (-0.13, 0.11)	-0.02 (-0.26, 0.22)	-0.05 (-0.24, 0.14)	-0.07 (-0.44, 0.30)
Social insurance	-0.09 (-0.20, 0.02)	-0.06 (-0.19, 0.07)	-0.12 (-0.43, 0.19)	-0.15 (-0.37, 0.07)	-0.26 (-0.73, 0.21)
Labour market protection	-0.03 (-0.35, 0.29)	0.03 (-0.35, 0.41)	0.25 (-0.26, 0.76)	-0.00 (-0.65, 0.64)	0.22 (-0.79, 1.23)
**Beneficiary incidence in Q1 (%)**					
Social assistance	-0.02 (-0.21, 0.16)	-0.23* (-0.44, -0.01)	-0.72*** (-1.13, -0.31)	-0.25 (-0.61, 0.11)	-0.91** (-1.57, -0.26)
Cash transfer	0.13 (-0.03, 0.29)	0.12 (-0.05, 0.28)	0.08 (-0.23, 0.40)	0.25 (-0.04, 0.53)	0.32 (-0.16, 0.80)
Social insurance	-0.15 (-0.30, 0.01)	-0.07 (-0.25, 0.12)	-0.16 (-0.59, 0.27)	-0.21 (-0.52, 0.10)	-0.35 (-1.01, 0.30)
Labour market protection	-0.14 (-0.29, 0.01)	-0.21* (-0.40, -0.02)	-0.15 (-0.37, 0.06)	-0.35 (-0.70, 0.01)	-0.49* (-0.90, -0.08)
**Social protection input per beneficiary (US dollars)**					
Social assistance	0.29 (-0.02, 0.60)	0.37 (-0.01, 0.75)	0.53 (-0.24, 1.31)	0.66 (-0.01, 1.32)	1.14 (-0.03, 2.30)
Cash transfer	0.02 (-0.47, 0.51)	0.30 (-0.19, 0.79)	4.09 (4.09, 4.09)	20.09 (20.09, 20.09)	23.75 (23.75, 23.75)
Social insurance	-0.02 (-0.23, 0.18)	0.09 (-0.14, 0.33)	0.14 (-0.42, 0.71)	0.07 (-0.33, 0.47)	0.19 (-0.67, 1.05)
Labour market protection	0.16 (-0.05, 0.36)	0.25 (-0.03, 0.53)	0.67 (-0.11, 1.45)	0.41 (-0.03, 0.84)	1.02 (-0.15, 2.19)
**Social protection input per beneficiary in Q1 (US dollars)**					
Social assistance	0.29 (-0.01, 0.59)	0.36 (-0.06, 0.78)	0.68 (-0.17, 1.54)	0.65 (-0.02, 1.32)	1.27 (-0.02, 2.56)
Cash transfer	7.00 (7.00, 7.00)	4.96 (4.96, 4.96)	2.44 (2.44, 2.44)	11.95 (11.95, 11.95)	14.13 (14.13, 14.13)
Social insurance	0.06 (-0.15, 0.27)	0.24 (-0.01, 0.48)	0.42 (-0.18, 1.01)	0.30 (-0.12, 0.71)	0.68 (-0.22, 1.57)
Labour market protection	0.19 (-0.03, 0.41)	-0.09** (-0.16, -0.02)	0.71 (-0.12, 1.54)	0.52 (-0.03, 1.07)	1.18 (-0.03, 2.38)

Note:

1. NMR: neonatal mortality rate, PMR: post-neonatal mortality rate, CMR: childhood mortality rate, IMR: infant mortality rate, U5MR: under-5 mortality rate

2. *** indicates *P* value<0.001; ** indicates *P* value<0.01; * indicates *P* value<0.05
3. When analyzing the association between social protection input per beneficiary and health indicators, we divided the values of the health indicators by 10. So the coefficient β could be interpreted as when the social protection input per beneficiary increased by 10%, the mortality rate would change by β per 1,000 live births.

Table S10. Sensitivity analysis on the association between social protection programmes and inequalities of mortality indicators using the inequality measurements of concentration index[1]

	All social protection and labour programmes	
	Concentration index	Reducing inequalities
**Social protection coverage (%)**		
NMR	0.07 (-0.03, 0.17)	No
PMR	0.03 (-0.09, 0.14)	No
CMR	0.05 (0.01, 0.10)	Yes
IMR	0.04 (-0.05, 0.14)	No
U5MR	0.04 (-0.05, 0.13)	No
**Social protection coverage in Q1 (%)**		
NMR	0.02 (-0.09, 0.12)	No
PMR	0.04 (-0.06, 0.14)	No
CMR	0.07 (0.01, 0.14)	Yes
IMR	0.05 (-0.03, 0.13)	No
U5MR	0.04 (-0.04, 0.12)	No
**Beneficiary incidence in Q1 (%)**		
NMR	0.14 (-0.15, 0.44)	No
PMR	0.23 (0.02, 0.44)	Yes
CMR	0.19 (0.02, 0.37)	Yes
IMR	-0.05 (-0.32, 0.23)	No
U5MR	-0.10 (-0.37, 0.16)	No
**Social protection input per beneficiary (US dollars)**		
NMR	0.20 (-0.22, 0.62)	No
PMR	0.27 (-0.19, 0.72)	No
CMR	0.33 (-0.16, 0.82)	No
IMR	0.21 (-0.17, 0.58)	No
U5MR	0.24 (-0.12, 0.60)	No
**Social protection input per beneficiary in Q1 (US dollars)**		
NMR	0.18 (-0.26, 0.63)	No
PMR	0.43 (-0.03, 0.90)	No
CMR	0.38 (-0.13, 0.88)	No
IMR	0.28 (-0.11, 0.68)	No
U5MR	0.31 (-0.07, 0.69)	No

Note:

1. NMR: neonatal mortality rate, PMR: post-neonatal mortality rate, CMR: childhood mortality rate, IMR: infant mortality rate, U5MR: under-5 mortality rate, No: no significant change

Table S11. Sensitivity analysis on the association between social protection programmes and inequalities of mortality indicators using the inequality measurements of slope index of inequality[1]

	All social protection and labour programmes	
	**Slope index of inequality**	**Reducing inequalities**
**Social protection coverage (%)**		
NMR	-0.06 (-0.20, 0.07)	No
PMR	0.11 (-0.02, 0.25)	No
CMR	0.18 (0.02, 0.34)	Yes
IMR	0.04 (-0.19, 0.27)	No
U5MR	0.21 (-0.15, 0.56)	No
**Social protection coverage in Q1 (%)**		
NMR	-0.07 (-0.18, 0.05)	No
PMR	0.09 (-0.03, 0.20)	No
CMR	0.20 (0.02, 0.38)	Yes
IMR	0.01 (-0.18, 0.21)	No
U5MR	0.17 (-0.13, 0.48)	No
**Beneficiary incidence in Q1 (%)**		
NMR	-0.13 (-0.51, 0.26)	No
PMR	0.54 (0.13, 0.94)	Yes
CMR	0.93 (0.29, 1.57)	Yes
IMR	0.22 (-0.44, 0.88)	No
U5MR	0.62 (-0.05, 1.30)	No
**Social protection input per beneficiary (US dollars)**		
NMR	0.26 (-0.25, 0.78)	No
PMR	0.07 (-0.43, 0.58)	No
CMR	0.02 (-0.92, 0.96)	No
IMR	0.37 (-0.43, 1.18)	No
U5MR	0.49 (-0.93, 1.90)	No
**Social protection input per beneficiary in Q1 (US dollars)**		
NMR	0.18 (-0.37, 0.73)	No
PMR	0.11 (-0.41, 0.64)	No
CMR	0.01 (-0.98, 0.99)	No
IMR	0.29 (-0.55, 1.13)	No
U5MR	0.32 (-1.15, 1.78)	No

Note:

1. NMR: neonatal mortality rate, PMR: post-neonatal mortality rate, CMR: childhood mortality rate, IMR: infant mortality rate, U5MR: under-5 mortality rate, No: no significant change

Table S12. Sensitivity analysis on the association between social protection programmes and inequalities of mortality indicators using the inequality measurements of difference between the richest (Q5) and the poorest (Q1) quintiles[1]

	All social protection and labour programmes	
	**Difference between Q5 and Q1**	**Change in equity**
**Social protection coverage (%)**		
NMR	-0.06 (-0.16, 0.04)	No
PMR	0.09 (-0.02, 0.20)	No
CMR	0.17 (0.00, 0.34)	Yes
IMR	0.03 (-0.15, 0.21)	No
U5MR	0.18 (-0.09, 0.46)	No
**Social protection coverage in Q1 (%)**		
NMR	-0.04 (-0.11, 0.03)	No
PMR	0.07 (-0.03, 0.17)	No
CMR	0.16 (0.02, 0.30)	Yes
IMR	0.01 (-0.15, 0.16)	No
U5MR	0.16 (-0.07, 0.40)	No
**Beneficiary incidence in Q1 (%)**		
NMR	-0.06 (-0.38, 0.26)	No
PMR	0.34 (0.00, 0.69)	Yes
CMR	0.75 (0.25, 1.26)	Yes
IMR	0.28 (-0.28, 0.85)	No
U5MR	0.97 (0.14, 1.81)	Yes
**Social protection input per beneficiary (US dollars)**		
NMR	0.09 (-0.32, 0.50)	No
PMR	0.15 (-0.29, 0.58)	No
CMR	0.04 (-0.70, 0.79)	No
IMR	0.24 (-0.44, 0.92)	No
U5MR	0.29 (-0.84, 1.43)	No
**Social protection input per beneficiary in Q1 (US dollars)**		
NMR	0.07 (-0.35, 0.50)	No
PMR	0.13 (-0.33, 0.58)	No
CMR	0.01 (-0.77, 0.79)	No
IMR	0.20 (-0.51, 0.91)	No
U5MR	0.23 (-0.96, 1.41)	No

Note:

1. NMR: neonatal mortality rate, PMR: post-neonatal mortality rate, CMR: childhood mortality rate, IMR: infant mortality rate, U5MR: under-5 mortality rate, No: no significant change
